# Pharmacological Effects of Wenjing Decoction in a Rat Model of Cold Coagulation and Blood Stasis Primary Dysmenorrhea: An Integrated Analysis of Serum Pharmacochemistry, Network Pharmacology and Metabolomics

**DOI:** 10.3390/ph19091385

**Published:** 2026-09-01

**Authors:** Junge Li, Yuxin Liu, Xin Shao, Yuanlu Zhang, Zhidong Qiu, Yongchun Wang, Feiran Qi, Qiuzhu Tang, Ailing Jia

**Affiliations:** School of Pharmacy, Changchun University of Chinese Medicine, Changchun 130117, China; lijunge5951@163.com (J.L.); 13689817653@163.com (Y.L.); shaoxinshaowei@163.com (X.S.); 15844675576@163.com (Y.Z.); qzdcczy@163.com (Z.Q.); 18846926574@163.com (Y.W.); 15630861203@163.com (F.Q.)

**Keywords:** Wenjing Decoction, Cold Coagulation and Blood Stasis Primary Dysmenorrhea, serum pharmacochemistry, metabolomics, network pharmacology

## Abstract

**Background**: Primary dysmenorrhea (PD) is a prevalent gynecological condition that significantly compromises the quality of life in adolescents and women of reproductive age. Within traditional Chinese medicine (TCM), Cold Coagulation and Blood Stasis Primary Dysmenorrhea (CCBS-PD) represents the most frequently observed syndrome pattern of PD. Wenjing Decoction (WJD), a classical TCM formulation, has been extensively employed for the treatment of CCBS-PD. However, given the multi-component and complex nature of WJD, the potential mechanisms underpinning its therapeutic effect have yet to be elucidated. **Methods**: A rat model of CCBS-PD was induced through ice-water bath stimulation in conjunction with estradiol benzoate and oxytocin. The pharmacological effects of WJD were evaluated by writhing response, hemorheological parameters, uterine index, histopathological examination, and biochemical assays. Serum-exposed constituents of WJD were characterized using UPLC-Orbitrap Exploris 120 MS. To study the mechanisms of WJD, methods from network pharmacology, molecular docking, and off-target metabolomics were used. Western blot analysis examined representative proteins in the signaling pathways predicted by network pharmacology. Network pharmacology and metabolomics were integrated to construct a pathway–metabolite–target–compound network, and representative targets were analyzed by RT-qPCR. **Results**: WJD treatment reduced writhing responses, improved hemorheological abnormalities, and alleviated uterine pathological changes in CCBS-PD rats. Serum pharmacochemistry identified 62 WJD-derived constituents. Metabolomics analysis indicated that WJD was associated with alterations in nitrogen metabolism, valine/leucine/isoleucine biosynthesis and arginine biosynthesis. Network pharmacology and molecular docking suggested several candidate compounds and targets, including Robinetin, Levistolide A, Pratol, 7,4′-dihydroxyflavone, EGFR, AKT1, ESR1, MMP9, MAPK3, and TNF. RT-qPCR showed that selected genes, including AKT1, EGFR, MAPK3, TNF, ESR1, MMP9 and CASP3, were changed in the model group and partially restored following WJD improvement. Western blot analysis demonstrated that WJD treatment decreased the phosphorylation levels of AKT, ERK1/2, and NF-κB, and down-regulated the protein expression of COX-2. **Conclusions**: WJD showed beneficial effects in a CCBS-PD rat model. Synthesized assessments indicate a potential link to the actions of serum-exposed constituents, alterations in amino acid metabolism, and modulation of inflammation-related signaling pathways. This research offers initial indications suggesting the multi-component and multi-target pharmacological actions of WJD, although the underlying mechanisms remain to be validated through targeted metabolomics and functional studies.

## 1. Introduction

Primary dysmenorrhea (PD) is a common gynecological disorder marked by pain and bloating in the lower abdomen that occurs during, just before, or shortly after menstruation, with no detectable structural abnormalities in the reproductive organs [1]. Common accompanying symptoms include back pain, fatigue, and nausea. PD predominantly affects adolescent and reproductive-age women, with reported prevalence rates ranging from 30% to 80% [2,3]. This condition may substantially affect individuals’ physical and psychological well-being, as well as their academic and occupational performance and quality of life [4]. In contemporary medicine, the pathogenesis of PD is attributed to aberrant prostaglandin synthesis, excessive contractions of uterine smooth muscle, disturbances in neuroendocrine function, and dysregulation of inflammatory responses [5,6]. Clinical symptomatic management commonly uses NSAIDs and oral contraceptives for rapid pain relief, but long-term use may lead to adverse effects including gastrointestinal irritation and drug resistance. Moreover, these treatments fail to target underlying physiological dysfunctions, restricting their long-term efficacy [7,8].

According to the principles of classical Chinese herbal medicine, PD is classified as menstrual abdominal pain [9]. The most common underlying cause, accounting for over 60% of cases, is cold stagnation and blood stasis syndrome [10]. This syndrome can result from external invasion by cold pathogens, internal injury from the ingestion of raw and cold foods, emotional disturbances, or constitutional cold deficiency. Its core pathogenesis involves stagnation of cold and impaired flow of qi and blood, leading to pain. Therapy aims to warm the meridians to expel cold, enhance blood circulation to resolve stasis, and regulate menstruation to relieve pain [11,12].

The classic Wenjing Decoction (WJD), documented by Chen Ziming in the Song Dynasty’s *Comprehensive Collection of Effective Formulas for Women* (*Fu Ren Da Quan Liang Fang*), serves as a representative formula used in the clinical management of gynecological disorders such as abdominal pain during menstruation and menstrual disorders of the cold coagulation and blood stasis type. It typically comprises nine herbal ingredients: *Angelica sinensis* (Oliv.) Diels, wine-processed (Jiudanggui, JDG); *Paeonia lactiflora* Pall. (Baishao, BS) and *Ligusticum chuanxiong* Hort (Chuanxiong, CX) for nourishing and activating blood; *Cinnamomum cassia* Presl (Rougui, RG) for warming meridians and dissipating cold; *Curcuma phaeocaulis* (Christm.) Roscoe, vinegar-processed (Cu’ezhu, CEZ) for breaking blood and promoting qi circulation; *Paeonia suffruticosa* Andr. (Mudanpi, MDP) for cooling blood and resolving stasis; *Panax ginseng* C.A. Mey. (Renshen, RS) and *Glycyrrhiza uralensis* Fisch., stir-fried licorice (Chaogancao, CGC) for reinforcing vital energy; and *Achyranthes bidentata* Blume, wine-processed (Jiuniuxi, JNX) for directing blood downward [13]. It exhibits the therapeutic actions of warming meridians, dissipating cold, activating blood, and unblocking meridians. Traditionally, this formula is indicated for conditions such as amenorrhea, dysmenorrhea, delayed menstruation, and lower abdominal pain resulting from cold pathogens invading the uterus and blood stasis. Owing to its well-documented clinical efficacy and long-standing application, WJD has become one of the classic prescriptions for regulating menstruation and alleviating pain in TCM gynecology. It is commonly used to treat CCBS-PD, demonstrating stable therapeutic efficacy, and the ability to holistically regulate qi, blood, yin, and yang [14]. Modern pharmacological research has confirmed WJD’s ability to relieve uterine smooth muscle spasms, reduce inflammation and pain, regulate hormones, and improve blood circulation [15]. However, the pharmacological characteristics of WJD in the treatment of CCBS-PD remain insufficiently defined, particularly regarding its serum-detected constituents and treatment-associated biological changes, which limits further interpretation and application.

Metabolomics, a core component of systems biology, captures comprehensive changes in endogenous metabolites during disease progression or drug intervention [16]. By identifying disease-specific metabolic biomarkers and differentially abundant metabolites following drug treatment, metabolomics can provide insights into disease-related metabolic alterations and potential therapeutic responses [17]. Serum pharmacochemistry is guided by the principle that blood-bound components represent potentially bioactive constituents [18]. By analyzing the serum-absorbed constituents following administration of TCM, candidate bioactive components associated with pharmacological effects can be identified [19]. Network pharmacology, an approach based on systems biology, constructs interaction networks among drugs, active components, targets, and diseases [20]. The integration of network pharmacology and metabolomics facilitates the analysis and visualization of the “pathway–metabolite–target–component” relationships, thereby revealing the potential underlying mechanisms of multi-component and multi-target characteristics of TCM formulas at a systems level [21,22].

Guided by the holistic theory of TCM and the multi-component nature of herbal formulas, this study assessed the pharmacological effects of WJD in a rat model of CCBS-PD. By integrating serum pharmacochemistry, network pharmacology, and metabolomics, we aimed to characterize serum-absorbed candidate components, evaluate WJD-associated metabolic changes, and explore potential component–target–pathway associations related to the observed pharmacological effects. These findings provide preliminary evidence and a systematic basis for further investigation of the potential mechanisms of WJD in CCBS-PD.

## 2. Results

### 2.1. Results of Body Weight, Writhing Response, Organ Index, and Hemorheology

**Figure 1:**
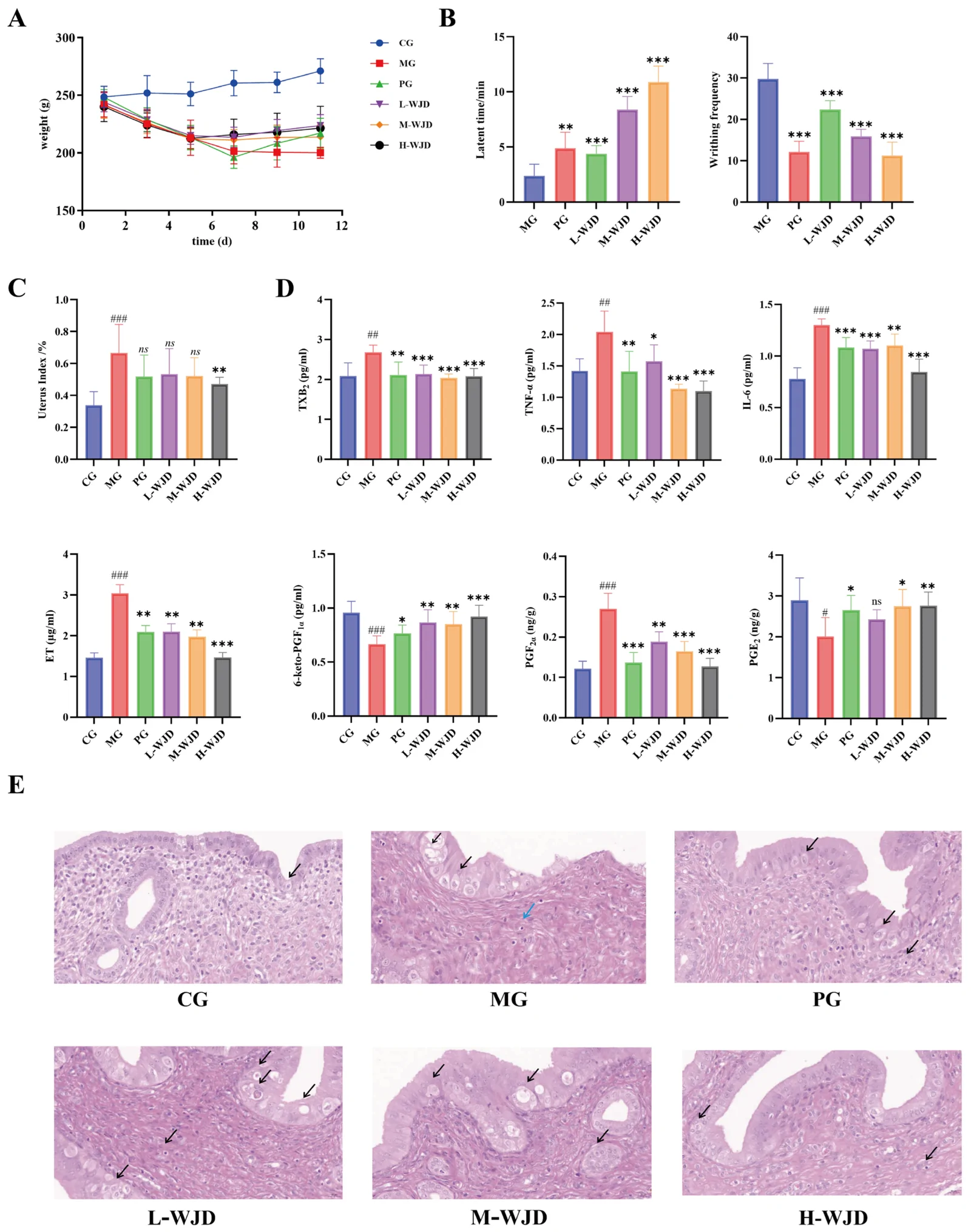
(**A**) Results of body weight changes (*n* = 8). (**B**) Writhing response results (*n* = 8). (**C**) Results of uterine index (*n* = 8). (**D**) Bar graph of serum factor expression levels in different groups of rats (*n* = 6). (**E**) HE-stained tissue sections (magnification: ×400, scale bar = 20 μm). Black arrows indicate cellular edema. Scattered inflammatory cell infiltration, predominantly lymphocytes, is observed (blue arrow). The data were expressed as the mean ± SD. ^#^ *p* < 0.05, ^##^ *p* < 0.01, ^###^ *p* < 0.001 vs. Control group; * *p* < 0.05, ** *p* < 0.01, *** *p* < 0.001 vs. Model group.

Body weight variations among rats in each group are shown in Figure 1A. Relative to the control group, the model group showed a marked reduction in body weight at the initial phase of the experiment, and this decreasing pattern continued over the entire observation period. In contrast, treatment with WJD mitigated weight loss to different extents, with the high-dose group demonstrating a more notable recovery.

The writhing response is a commonly used behavioral indicator for evaluating visceral pain-like responses in experimental dysmenorrhea models. In this study, no writhing responses were observed in the CG, which did not receive oxytocin stimulation, whereas rats in the model-related groups exhibited writhing behavior after oxytocin challenge (Figure 1B). Compared with the MG, both the PG and WJD-treated groups showed prolonged writhing latency and reduced writhing frequency. The writhing inhibition rate was increased in the WJD groups (Supplementary Table S2).

The uterine index was used as a gross indicator of uterine changes in the CCBS-PD model. As shown in Figure 1C and Supplementary Table S3, the uterine index was markedly elevated in the MG compared to the CG (*p* < 0.001). In comparison with the MG, treatment groups showed decreased uterine index values, with a significant reduction noted in the H-WJD group (*p* < 0.01). Nevertheless, the uterine index alone provides limited information and should be interpreted together with histopathological findings, inflammatory indicators, hemorheological parameters, and other pharmacodynamic observations.

TCM theory suggests that blood stasis syndrome is closely related to hemorheological alterations, such as impaired blood flow and increased blood viscosity. Whole-blood and plasma viscosities were measured to evaluate hemorheological changes in the CCBS-PD model (Table 1). Compared with the CG, the MG exhibited significantly higher low-shear whole-blood viscosity (L-WBV), medium-shear whole-blood viscosity (M-WBV), high-shear whole-blood viscosity (H-WBV), and plasma viscosity (PV, *p* < 0.01), suggesting the existence of hemorheological abnormalities in the model group, which are consistent with the characteristics of cold coagulation and blood stasis. Compared with the MG, the PG, L-WJD, M-WJD, and H-WJD groups showed significant reductions in whole-blood and plasma viscosities (*p* < 0.05). These results indicate that WJD administration might be associated with amelioration of hemorheological abnormalities in CCBS-PD model rats.

### 2.2. Serum Biochemistry and Histopathological Analysis

Evaluation of TXB_2_, 6-keto-PGF_1_α, ET, IL-6, TNF-α, PGF2α and PGE2 showed that WJD treatment significantly attenuated the alterations in serum and uterine inflammatory and vasoactive mediators observed in the model group (Figure 1D). Notably, the low-dose WJD group exhibited changes similar to those seen in the positive control group.

Histopathological examination of rat uterine tissue (Figure 1E), together with quantitative scores of stromal inflammatory infiltration and tissue edemas (Supplementary Table S4), showed clear morphological differences among groups. The CG displayed a normal endometrial structure without obvious pathological changes. In contrast, the MG exhibited endometrial thickening, epithelial cell swelling, vacuolar degeneration, partial epithelial shedding, and mild inflammatory cell infiltration, accompanied by significantly increased histopathological scores. WJD treatment mitigated these pathological alterations to varying extents, with the most notable effects seen in the H-WJD, which was consistent with the improvement in histopathological scores. Overall, WJD was associated with attenuation of uterine histopathological abnormalities in CCBS-PD model rats.

### 2.3. Untargeted Serum Metabolomics Results

#### 2.3.1. Multivariate Statistical Analysis

**Figure 2:**
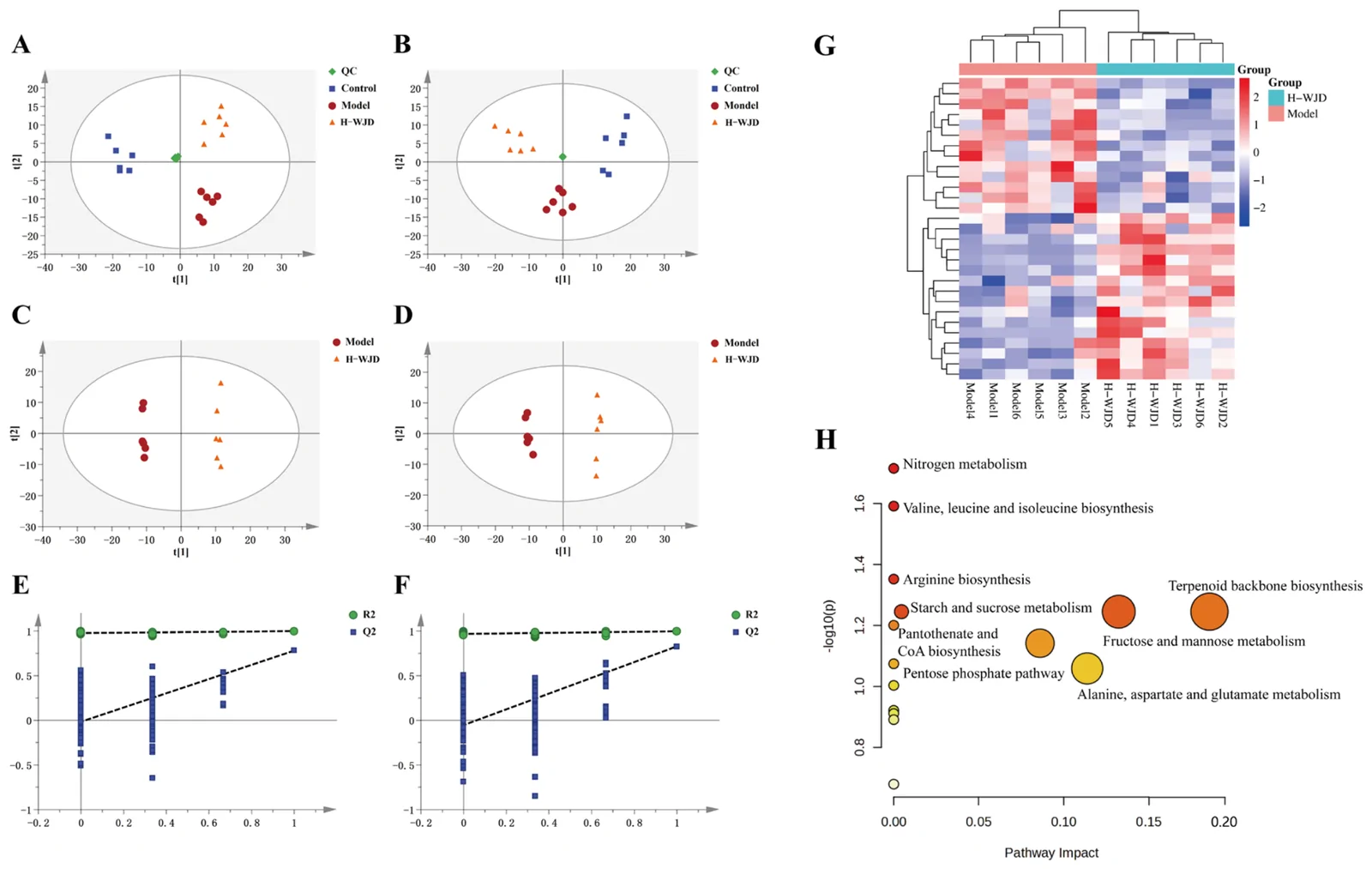
PCA score plots of serum samples from the Control, Model, Wenjing Decoction (H-WJD), and quality control (QC) groups in positive ion mode (**A**) and negative ion mode (**B**) OPLS-DA score plots of the Model group versus WJD group in serum samples under positive ion mode (**C**) and negative ion mode (**D**) Validation of the OPLS-DA model (Model vs. H-WJD) by 200 permutation tests in serum samples under positive ion mode (**E**) and negative ion mode (**F**). (**G**) Heat map of differentially abundant metabolites in serum. The color gradient from blue to red corresponds to a gradual increase in metabolite abundance. (**H**) Enriched metabolic pathways of differentially abundant serum metabolites.

To explore systemic metabolic alterations in CCBS-PD and those linked to WJD, untargeted metabolomics was carried out on serum samples from the CG, MG, and H-WJD groups using UPLC-Orbitrap Exploris 120 MS. Figure S1 in the Supplementary Materials displays the total ion current chromatograms acquired under both positive and negative ionization modes. Principal component analysis (PCA) was employed to illustrate global metabolic differences and identify possible outliers. The PCA score plots revealed clear within-group clustering and distinct separation between the CG and MG, as well as between the MG and H-WJD groups, reflecting metabolic profile differences among the groups. (Figure 2A,B). Subsequently, orthogonal partial least squares discriminant analysis (OPLS-DA) was employed to further assess group separation (Figure 2C,D). Separation trends were observed in the score plots. The stability of the model was assessed through 200 permutation tests, suggesting that the model showed acceptable stability and was not overfitted (Figure 2E,F).

#### 2.3.2. Identification and Exploratory Analysis of Candidate Differential Serum Metabolites

Candidate differentially abundant serum metabolites were screened based on comparisons among groups. A total of 76 candidate differentially abundant metabolites were detected between the MG and CG, including 29 upregulated and 47 downregulated metabolites (Supplementary Table S6). of which four remained significant after FDR correction. In addition, 29 candidate differentially abundant metabolites were detected between the MG and WJD groups, including 16 upregulated and 13 downregulated metabolites (Supplementary Table S7). After FDR correction, only 4-vinylphenol sulfate remained significant. Hierarchical clustering heatmap analysis was conducted on the candidate differentially abundant metabolites between the Model group and H-WJD group (Figure 2G). The results showed distinct clustering patterns between the two groups, and H-WJD treatment showed a tendency to reverse some of the metabolic alterations observed in the model group. These findings were interpreted as exploratory.

#### 2.3.3. Metabolic Pathway Analysis

To identify functional pathways associated with WJD-induced metabolic changes, exploratory pathway enrichment analysis was performed on the 29 candidate differentially abundant serum metabolites (WJD vs. MG) using MetaboAnalyst 5.0. A total of 15 metabolic pathways were identified (Figure 2H, Supplementary Table S8). Among these, nitrogen metabolism, valine–leucine–isoleucine biosynthesis, and arginine biosynthesis showed nominally significant enrichment (*p* < 0.05), whereas the remaining pathways showed suggestive enrichment.

### 2.4. Identification of Serum-Absorbed Constituents of WJD

**Figure 3:**
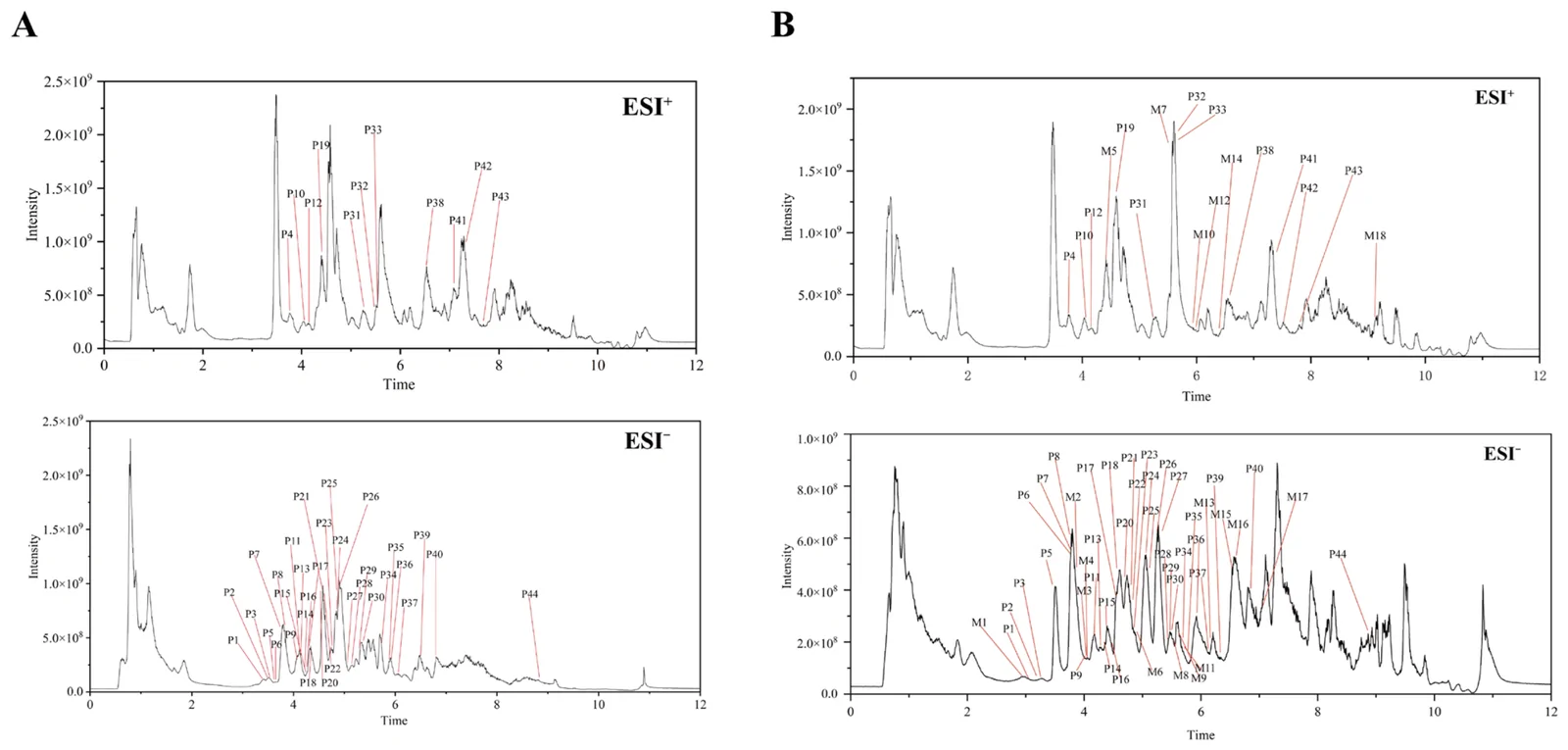
(**A**) Total ion chromatograms (TIC) of WJD prototype components in serum; (**B**) TIC of WJD in serum. P represents prototype components, while M represents metabolic components. Both were acquired using UPLC-Orbitrap Exploris 120 MS.

In accordance with the principles of serum pharmacochemistry, serum samples from both the CG and WJD-treated rats were subjected to analysis using UPLC-Orbitrap Exploris 120 mass spectrometry in positive and negative ionization modes (Figure 3). A total of 44 parent compounds and 18 metabolites were identified in the serum of WJD-treated rats (Supplementary Tables S9 and S10).

### 2.5. Network Pharmacology Analysis

#### 2.5.1. Identification of Intersection Targets and PPI Network Construction

**Figure 4:**
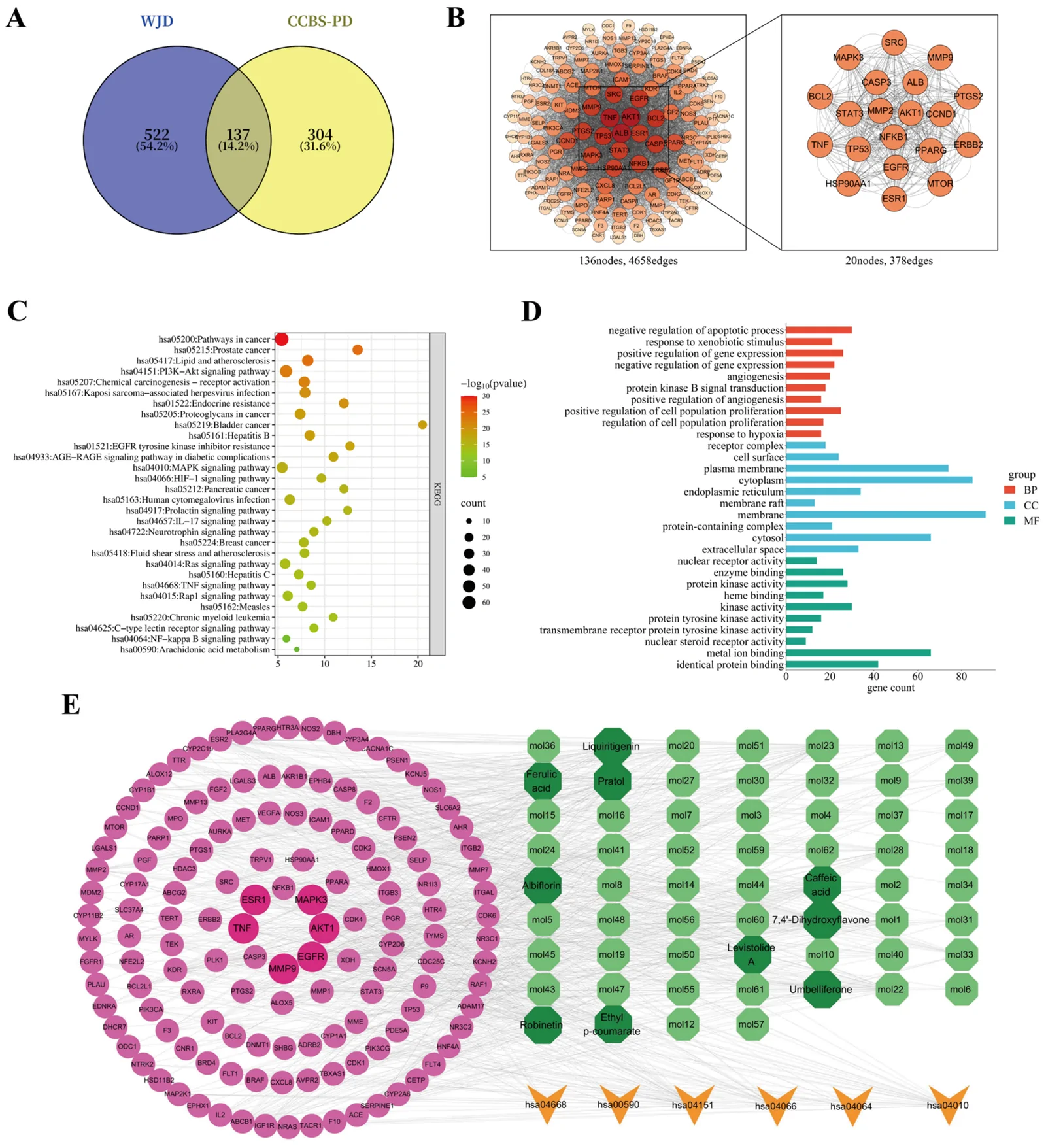
Network pharmacology analysis of WJD against CCBS-PD. (**A**) Venn diagram of the intersection of the targets between WJD and CCBS-PD. (**B**) Screening of key targets by relative topological parameters in PPI network. (**C**) GO enrichment analysis terms of 137 common targets. The top 10 terms of biological process, cellular component, and molecular function are displayed. (**D**) KEGG pathway enrichment analysis of 137 common targets. The 30 pathways are listed based on the *p* values. Node size is based on gene ratio; node color is based on −log (*p*) value. (**E**) Network diagram of Compound-Target-Pathway, mol represents components. Pathways are shown as orange “V” symbols; key genes, pink circles; WJD compounds, green hexagons. Darker node color indicates a higher degree value.

A total of 62 serum-absorbed constituents of WJD were identified based on serum pharmacochemistry analysis, and 659 corresponding putative targets were obtained through target prediction. Meanwhile, 441 CCBS-PD-related targets were retrieved from the GeneCards and DrugBank databases after removal of duplicates. All genes were standardized to Gene Symbols. Intersection analysis identified 137 overlapping targets potentially associated with WJD intervention in CCBS-PD (Figure 4A). To explore potential interactions, a PPI network was constructed using the STRING database (combined score > 0.4, without disconnected nodes) and visualized in Cytoscape for topological analysis. Following the elimination of isolated nodes, a refined network comprising 136 nodes was obtained. Key nodes were selected based on topological parameters including degree and eigenvector centrality, and 20 hub genes were identified for further analysis (Figure 4B).

#### 2.5.2. GO and KEGG Enrichment Analysis

Functional annotation of the 137 overlapping targets was conducted using GO and KEGG pathway enrichment analyses, with a significance cutoff of FDR < 0.05. This approach yielded 383 significantly enriched GO terms, distributed across 275 biological processes, 37 cellular components, and 71 molecular functions (Figure 4C). KEGG pathway enrichment analysis identified 155 significantly enriched pathways. Based on *p*-value ranking, 30 representative pathways were selected for visualization (Figure 4D). Among these, several pathways, including NF-κB, MAPK, PI3K-Akt, HIF-1, TNF and arachidonic acid metabolism pathway, showed relatively higher enrichment levels.

#### 2.5.3. Screening of Candidate Components and Core Targets

A compound–target–pathway (C-T-P) network was constructed using Cytoscape 3.10.3 to illustrate the relationships among the identified compounds, targets, and associated pathways (Figure 4E). The network comprised 62 compounds, 137 targets, and 6 signaling pathways. Topological analysis showed that 10 compounds exhibited relatively higher degree values, including 7,4′-Dihydroxyflavone, Pratol, Robinetin, Liquiritigenin, Ethyl p-coumarate, Albiflorin, Ferulic acid, Caffeic acid, Levistolide A, and Umbelliferone. These components were therefore considered highly connected compounds within the network. To further characterize the network features, an overlap analysis was performed between highly connected nodes from the PPI network and the top 30% of targets in the C-T-P network. This analysis identified six overlapping targets that may be linked to the observed effects of WJD in CCBS-PD, including EGFR, AKT1, MMP9, ESR1, MAPK3, and TNF.

### 2.6. Molecular Docking and Binding Trend Analysis of Candidate Components

**Figure 5:**
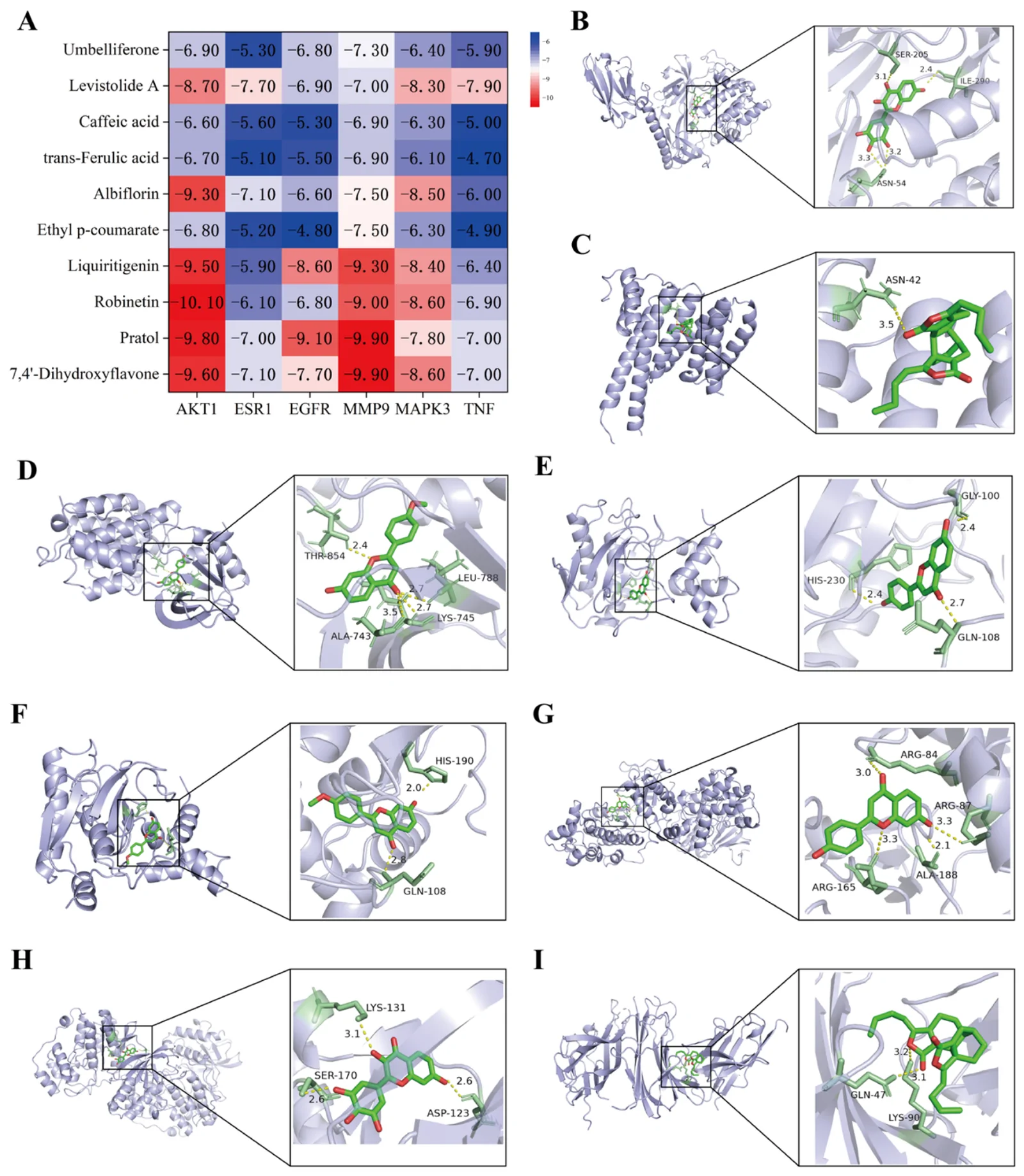
(**A**) Heat maps of molecular docking results. The redder the color, the smaller the corresponding value, indicating the stronger the docking strength. Docking patterns of six core targets and specific compounds. (**B**) Robinetin–AKT1 complex. (**C**) Levistolide A–ESR1 complex. (**D**) Pratol–EGFR complex. (**E**) 7,4′-Dihydroxyflavone–MMP9 complex. (**F**) Pratol–MMP9 complex. (**G**) 7,4′-Dihydroxyflavone–MAPK3 complex. (**H**) Robinetin–MAPK3 complex. (**I**) Levistolide A–TNF complex.

AutoDock Vina 1.5.7 was employed to perform molecular docking, aiming to explore potential interactions between the selected WJD compounds and the overlapping targets identified through network analysis. Docking energies were calculated to evaluate the relative favorability of the predicted ligand–target interactions; lower docking energies generally indicate more favorable predicted interactions (Figure 5A). The ligand–target pairs with relatively lower predicted binding energies included Robinetin–AKT1 (−10.10 kcal/mol), Levistolide A–ESR1 (−7.70 kcal/mol), Pratol–EGFR (−9.10 kcal/mol), 7,4′-Dihydroxyflavone–MMP9 (−9.90 kcal/mol), Pratol–MMP9 (−9.90 kcal/mol), 7,4′-Dihydroxyflavone–MAPK3 (−8.60 kcal/mol), Robinetin–MAPK3 (−8.60 kcal/mol), and Levistolide A–TNF (−7.90 kcal/mol). These results suggest that the selected compounds may have favorable predicted interactions with the corresponding targets. Representative binding modes for these compound–target pairs are shown in Figure 5. However, molecular docking provides only in silico predictive evidence and does not constitute direct evidence of physical ligand–target binding. Further experimental studies are therefore required to verify these predicted interactions and establish their biological relevance.

### 2.7. Effects of WJD on the Major Signaling Pathways and Related Proteins

**Figure 6:**
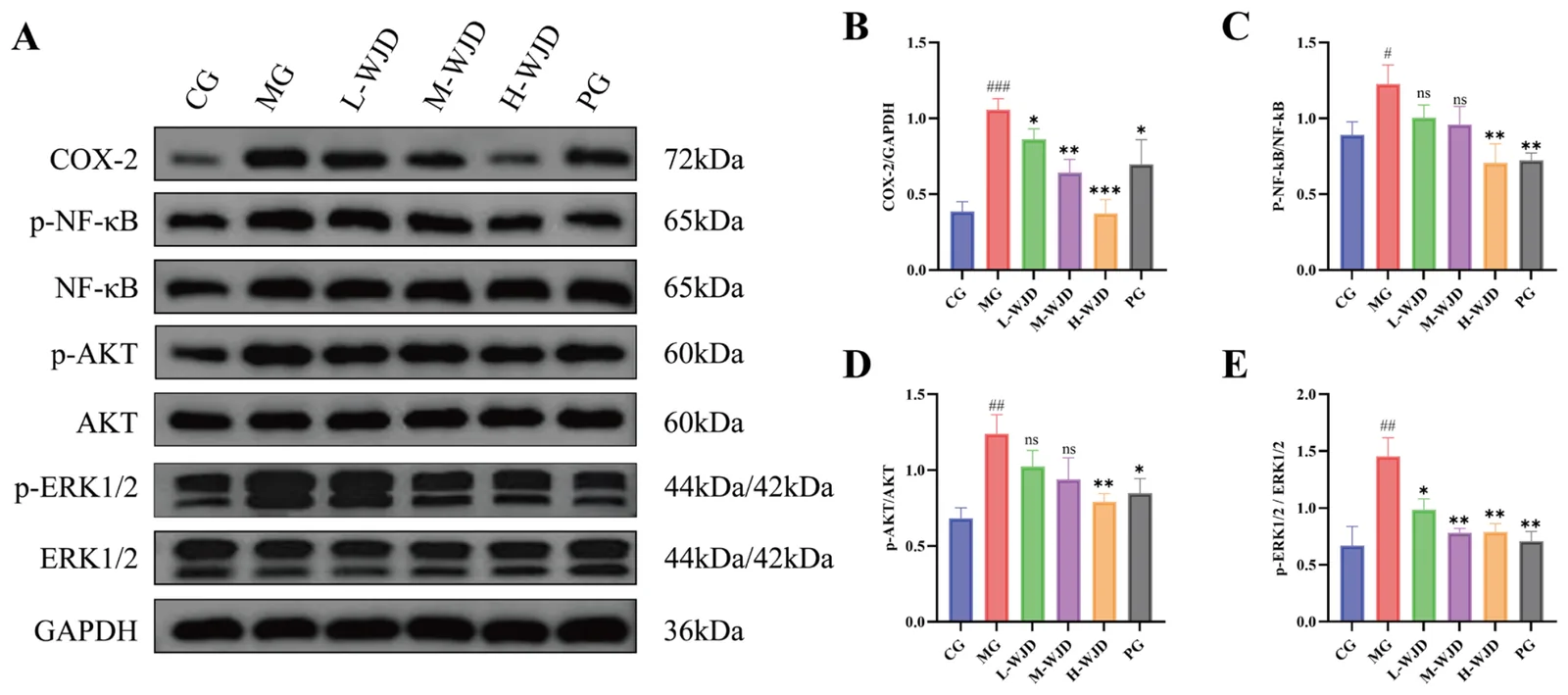
(**A**) Representative Western blot images showing protein expression levels of COX-2, p-NF-κB, NF-κB, p-AKT, AKT, p-ERK1/2, ERK1/2, and GAPDH (loading control) in different groups. (**B**–**E**) Quantitative analysis of protein expression levels normalized to GAPDH: (**B**) COX-2, (**C**) p-NF-κB/NF-κB, (**D**) p-AKT/AKT, (**E**) p-ERK1/2/ERK1/2. The data were expressed as the mean ± SD (*n* = 3). ns, not significant. ^#^ *p* < 0.05, ^##^ *p* < 0.01, ^###^ *p* < 0.001 vs. Control group; * *p* < 0.05, ** *p* < 0.01, *** *p* < 0.001 vs. Model group.

Leveraging the integrated results of network pharmacology enrichment and molecular docking, key signaling pathways and their associated proteins were identified for further investigation. As illustrated in Figure 6, compared with the CG, the MG showed markedly increased expression ratios of p-AKT/AKT, p-ERK1/2/ERK1/2 and p-NF-κB/NF-κB, alongside markedly upregulated COX-2 protein expression. Following WJD treatment, the phosphorylation levels of the aforementioned proteins and COX-2 expression were attenuated to varying degrees. By integrating network pharmacology predictions with experimental validation, these data indicate that WJD may partially alleviate inflammatory responses and ameliorate the pathological manifestations of primary dysmenorrhea—particularly those associated with cold coagulation and blood stasis syndrome—primarily through the suppression of key signaling cascades, including PI3K/AKT, MAPK, and NF-κB.

### 2.8. Integrated Analysis of Metabolomics and Network Pharmacology in CCBS-PD

**Figure 7:**
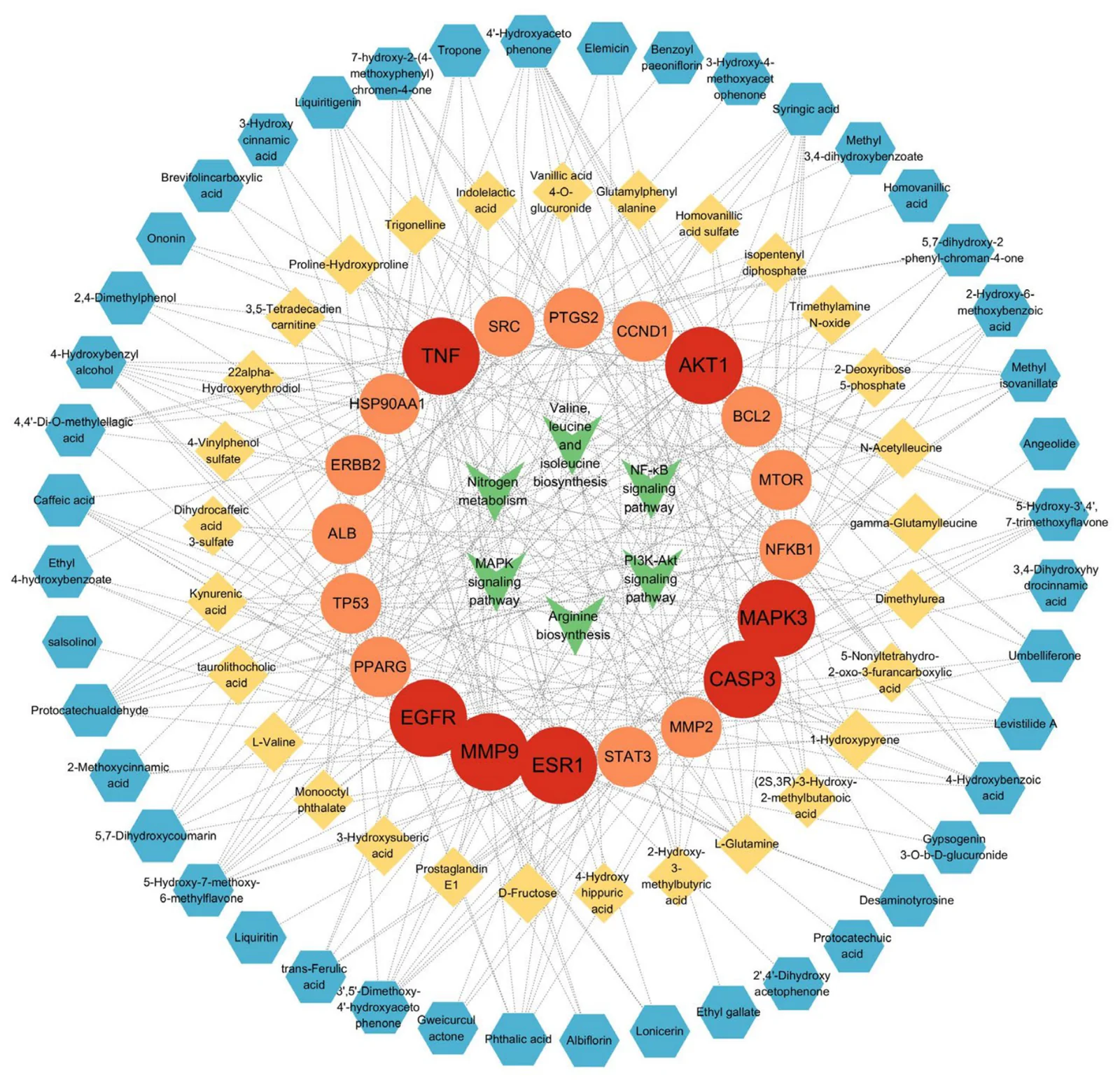
Integration of the results from metabolomics and network pharmacology to construct a “pathway–metabolite–target–compound” network. Pathways are shown as green “V” symbols; key genes, red circles; metabolites, yellow diamonds; WJD compounds, blue hexagons.

**Figure 8:**
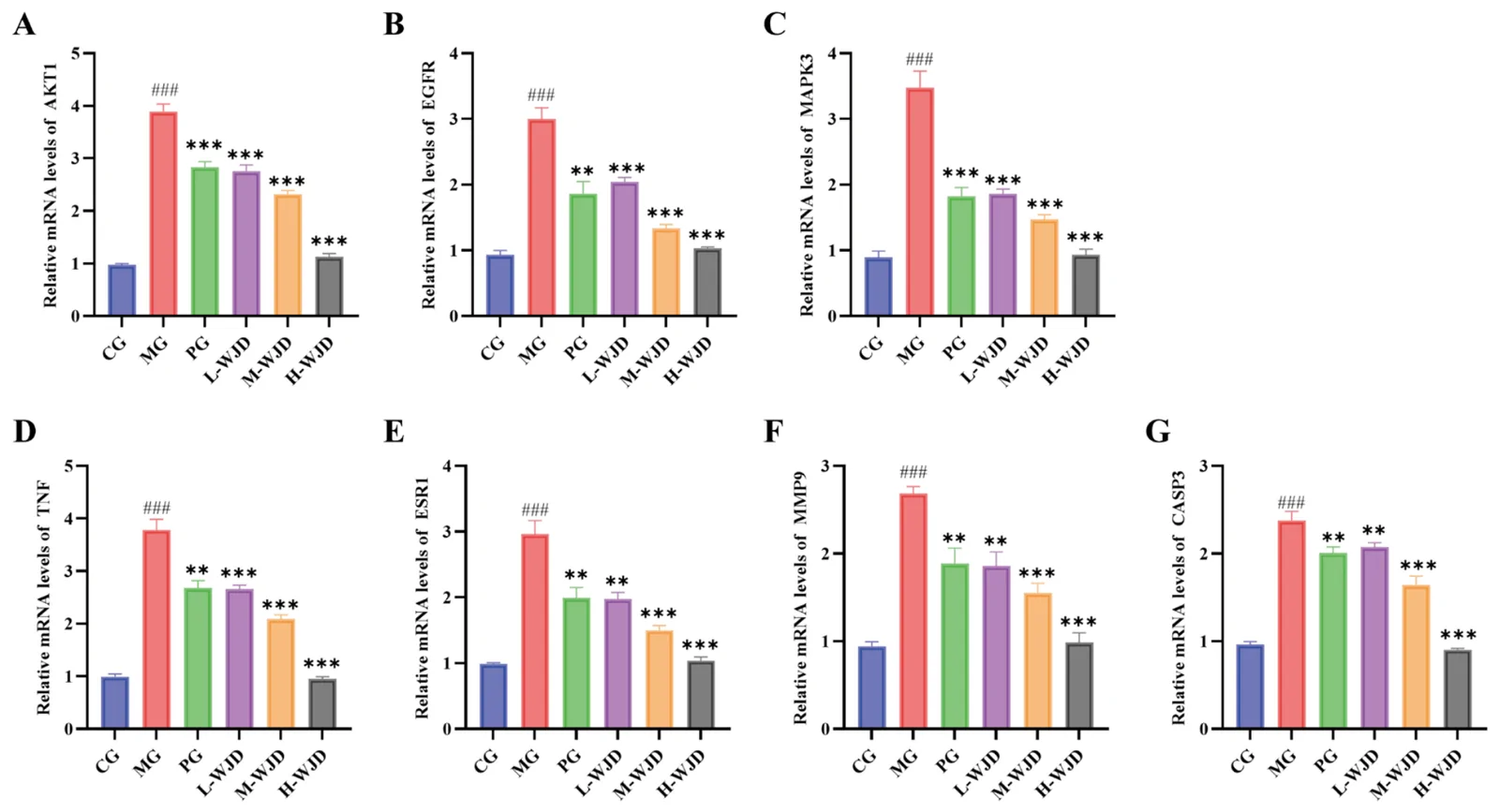
The results of experimental verification in vivo. Relative mRNA expression levels of (**A**) AKT1, (**B**) EGFR, (**C**) MAPK3, (**D**) TNF, (**E**) ESR1, (**F**) MMP9 and (**G**) CASP3. The data were expressed as the mean ± SD (*n* = 3). ^###^ *p* < 0.001 vs. Control group; ** *p* < 0.01, *** *p* < 0.001 vs. Model group.

To identify biologically relevant signaling cascades, priority was given to significantly enriched KEGG pathways that incorporated key molecular docking targets and aligned with primary dysmenorrhea pathophysiology. A multi-omics integration strategy combining metabolomic profiling and network pharmacology was employed to construct a “pathway–metabolite–target–compound” interactome encompassing 6 pathways, 20 hub genes, 29 metabolites, and 44 phytochemicals, thereby elucidating the potential mechanisms of WJD in treating CCBS-PD. From this integrated framework, AKT1, EGFR, MAPK3, TNF, ESR1, MMP9, and CASP3 emerged as candidate key targets (Figure 7). These targets were recognized as metabolite-associated responsive genes rather than merely predicted targets. Their relative mRNA expression levels were then measured using RT-qPCR. (Figure 8). Relative to the CG, the MG exhibited significantly elevated mRNA expression levels of AKT1, EGFR, MAPK3, TNF, ESR1, MMP9, and CASP3. After WJD administration, the expression of these genes was markedly reduced. Collectively, these findings imply that WJD orchestrates the expression of pivotal genes, including AKT1, EGFR, MAPK3, TNF, ESR1, MMP9, and CASP3, potentially modulating both metabolic dysregulation and inflammatory cascades characteristic of CCBS-PD.

## 3. Discussion

This work explores the therapeutic actions of WJD in a CCBS-PD rat model using pharmacological assessment combined with serum pharmacochemistry, metabolomics, and network-based analysis. The established model exhibited typical dysmenorrhea-like features, including increased writhing response, hemorheological disturbances, and inflammatory activation. WJD administration alleviated these abnormalities, suggesting overall beneficial effects under the present experimental conditions.

Pharmacodynamic results showed that WJD reduced writhing responses, improved hemorheological parameters, and alleviated uterine pathological injury. In addition, WJD modulated several vasoactive and inflammatory mediators, including TXB_2_, 6-keto-PGF_1_α, ET, IL-6, TNF-α, PGF_2_α, and PGE_2_, suggesting an association with improved blood flow, regulation of prostaglandin balance, and attenuation of inflammatory responses in the CCBS-PD model [23]. In the present study, WJD treatment was associated with decreased TXB_2_, ET, IL-6, TNF-α, and PGF_2_α levels, together with increased 6-keto-PGF_1_α and PGE_2_ levels, indicating a shift toward improved vascular and inflammatory homeostasis. TXA_2_ and PGI_2_ are key regulators of vascular tone, and their imbalance contributes to vasospasm and hypercoagulability [24]. The observed changes in their stable metabolites (TXB_2_ and 6-keto-PGF_1_α) may reflect partial restoration of this balance. In addition, ET elevation is associated with reduced uterine perfusion [25], while IL-6 and TNF-α promote inflammatory responses and prostaglandin synthesis [26]. The reduction in these factors after WJD treatment suggests a potential attenuation of inflammation-related processes. Overall, these findings indicate that WJD may be linked to the coordinated regulation of prostaglandin metabolism, vasoactive balance, and inflammatory responses, although the underlying mechanisms require further investigation.

Untargeted metabolomics analysis revealed that WJD intervention was associated with changes in the serum metabolic profile of CCBS-PD rats, with nitrogen metabolism, valine/leucine/isoleucine biosynthesis, and arginine biosynthesis identified as the main enriched pathways. These pathways are largely related to amino acid metabolism, energy homeostasis, and vascular regulation. Nitrogen metabolism has been reported to be associated with ischemia- and hypoxia-related conditions [27], which may be relevant to the reduced local blood flow caused by cold exposure and uterine contraction in this model. Alterations in the glutamate–glutamine cycle may affect glutamine and glutamate homeostasis [28], while glutamine also serves as an important nitrogen donor and precursor for glutathione synthesis [29]; suggesting a possible link between nitrogen metabolism and stress adaptation. Branched-chain amino acids, including valine, leucine, and isoleucine, participate in energy metabolism and may contribute to ATP production under metabolic stress [30], partly through metabolic processes involving branched-chain amino acid transaminases, glutamate dehydrogenase, and glutamate-related intermediates [31]. In addition, arginine metabolism is closely related to nitric oxide production, vascular tone, and microcirculatory regulation [32,33] and its metabolic connection with glutamate and glutamine suggests potential crosstalk between nitrogen metabolism and arginine-related pathways [34]. Collectively, these findings indicate that WJD treatment may be associated with coordinated changes in amino acid metabolism, energy adaptation, and vascular-related metabolic regulation in CCBS-PD. However, these interpretations are based on untargeted metabolomics and exploratory pathway enrichment analysis, and should therefore be interpreted as exploratory, requiring further validation by targeted metabolomics and functional studies.

Network pharmacology and molecular docking analyses initially pinpointed multiple candidate compounds and putative targets linked to WJD in CCBS-PD. Core targets, including EGFR, AKT1, MAPK3, TNF, ESR1, and MMP9, were mainly enriched in pathways such as PI3K/AKT, MAPK, NF-κB, TNF, HIF-1, and arachidonic acid metabolism, which are related to inflammatory responses, vascular regulation, and prostaglandin metabolism. Among the predicted compounds, Robinetin, 7,4′-dihydroxyflavone, Levistolide A, and Pratol showed relatively favorable binding affinities with several key targets, suggesting that they may serve as candidate compounds for further investigation. Previous studies have reported that these compounds exhibit anti-inflammatory, antioxidant, and apoptosis-related activities, often involving pathways such as NF-κB, PI3K/AKT, MAPK, and TLR4, along with regulation of cytokines and oxidative stress-related factors [35,36,37,38,39,40,41,42,43].

Under conditions of cold stagnation and blood stasis, uterine ischemia, hypoxia, and inflammatory responses are considered key pathological features of dysmenorrhea. Previous studies have shown that TNF can activate NF-κB and MAPK signaling pathways [44,45], while MAPK3 may regulate downstream genes involved in inflammation and extracellular matrix remodeling [46]. In addition, the PI3K–AKT pathway is closely associated with cell survival and apoptosis, and can be influenced by EGFR-mediated signaling [47,48]. Estrogen signaling, particularly via ESR1, has also been implicated in uterine contractility, vascular function, and inflammatory regulation through interactions with PI3K–AKT and MAPK pathways [49,50]. COX-2 is a key enzyme in arachidonic acid metabolism, catalyzing the conversion of arachidonic acid into prostaglandin precursors. Moreover, it plays a critical role in the production of pro-inflammatory mediators [51,52].

To further evaluate the biological relevance of the pathways identified by network pharmacology, Western blot analysis was performed. Phosphorylation of AKT, ERK1/2, and NF-κB, together with COX-2 expression, was elevated in the MG and attenuated following WJD treatment. These alterations were largely in agreement with the pathway enrichment findings, reinforcing the likely role of PI3K/AKT, MAPK, and NF-κB signaling in the observed pharmacological effects. Moreover, COX-2, a key enzyme in arachidonic acid metabolism that converts arachidonic acid into prostaglandin precursors, thereby promoting the production of pro-inflammatory mediators, was similarly downregulated after WJD administration, suggesting a possible link between altered inflammatory signaling and the metabolomic perturbations observed in arachidonic acid metabolism. In parallel, integrated analysis combining metabolomics and network pharmacology revealed several metabolite-associated responsive genes, including AKT1, EGFR, MAPK3, TNF, ESR1, MMP9, and CASP3. RT-qPCR showed that the expression of these genes exhibited changes consistent with both the protein-level observations and the computational predictions. Collectively, these findings provide convergent but associative evidence that WJD may be linked to coordinated modulation of inflammation-related signaling pathways and metabolic alterations under the present experimental conditions, although the causal relationships between these molecular events remain to be further established.

**Figure 9:**
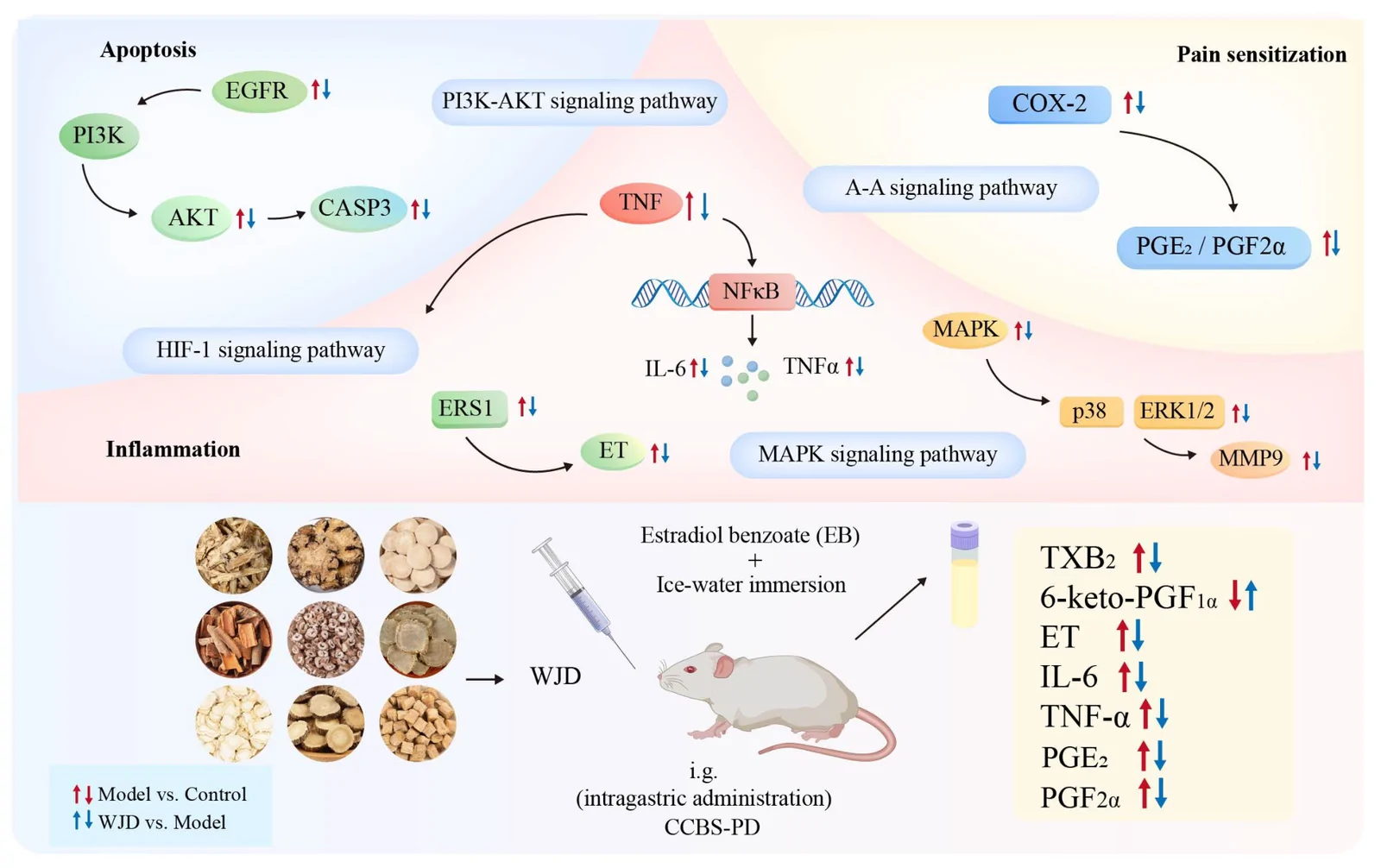
Schematic diagram of the potential mechanisms of WJD in CCBS-PD.

Building on the integrated analyses and experimental validation described above, the effects of WJD on CCBS-PD may be associated with coordinated regulation of hemorheological status, inflammatory mediators, prostaglandin-related balance, amino acid metabolism, and inflammation-associated signaling pathways (Figure 9). Nevertheless, the present findings rely largely on correlative evidence. Network pharmacology and molecular docking offer predictive insights, whereas RT-qPCR and Western blot analyses indicate alterations at the expression level rather than direct causal regulation.

Several limitations should be acknowledged. Serum compounds were mainly identified qualitatively, and quantitative characterization of key constituents is still needed. In addition, the metabolomics results require further validation using targeted approaches. Although gene and protein expression analyses provided supportive evidence, they do not fully establish direct target engagement or functional pathway regulation. Moreover, the animal model may not fully capture the complexity of human primary dysmenorrhea, and species-related differences in target regulation and signaling responses should be considered when extrapolating these findings to humans.

Taken together, the current study offers initial evidence suggesting a potential association between WJD multi-component and multi-target effects involving inflammatory regulation, prostaglandin-related balance, vascular function, and metabolic modulation in CCBS-PD, which may serve as a basis for future in-depth investigations.

## 4. Materials and Methods

### 4.1. Medicinal Materials and Reagents

Rougui (dried cortex of *Cinnamomum cassia* Presl, RG, collected in Guangxi Yulin, no. 2405093), Renshen (dried radix and rhizoma of *Panax ginseng* C.A.Mey, RS, collected in Jilin Tonghua, no. 2406072), Jiudanggui (processed radix of *Angelica sinensis* (Oliv.) Diels with wine, JDG, collected in Gansu Minxian, no. 2408012), Chuanxiong (dried rhizoma of *Ligusticum chuanxiong* Hort, CX, collected in Sichuan Pengzhou, no. 2409211), Baishao (dried radix of *Paeonia lactiflora* Pall, BS, collected in Anhui Bozhou, no. 2406038), Mudanpi (dried root bark of *Paeonia suffruticosa* Andr, MDP, collected in Anhui Tongling, no. 2404061), Cu’ezhu (processed rhizoma of *Curcuma phaeocaulis* Val. with vinegar, CEZ, collected in Guangxi Yulin, no. 2406064), Jiuniuxi (processed radix of *Achyranthes bidentata* Bl. with wine, JNX, collected in Henan Jiaozuo, no. 2405102), Chaogancao (processed radix and rhizoma of *Glycyrrhiza uralensis* Fisch. by stir-frying, CGC, collected in Xinjiang Hetian, no. 2404062). All herbal materials were purchased from Jilin Beiyao Chinese Medicine Pharmaceutical Group Co., Ltd., Changchun, China. All herbal materials were identified by professional traditional Chinese medicine specialists and used only after passing quality inspection in strict accordance with Volume I of the 2020 edition of Pharmacopoeia of the People’s Republic of China. Rat endothelin-1 (ET-1) ELISA Kit, Rat thromboxane B2 (TXB2) ELISA Kit, Rat 6-keto-prostaglandin F1α (6-keto-PGF1α) ELISA Kit, Rat interleukin-6 (IL-6) ELISA Kit, Rat tumor necrosis factor-α (TNF-α), Rat Prostaglandin F_2_α (PGF_2_α) and Rat Prostaglandin E_2_ (PGE2) ELISA Kit were purchased from Jiangsu Meimian Industrial Co., Ltd. (Yancheng, China), with the catalog numbers MM-0634R1, MM-0516R1, MM-0268R1, MM-0190R1, MM-0180R1, MM-0230R1 and MM-0068R1 respectively. Estradiol Benzoate Injection (Ningbo No.2 Hormone Factory, Ningbo, China, C2405161); Oxytocin Injection (Ningbo No.2 Hormone Factory, Ningbo, China, C2401241); Normal Saline (Harbin Sanlian Pharmaceutical Co., Ltd., Harbin, China, 240821W01); Tongjingbao Granules (Zhongjing Wanxi Pharmaceutical Co., Ltd., Nanyang, China, 131017); Urethane (Yuanye, Shanghai, China, S11036) were also used. Additionally, 4% paraformaldehyde general-purpose tissue fixative was purchased from Biosharp (Beijing, China), Cat.BL539A. Methanol, acetonitrile, isopropanol, and acetic acid (all MS grade) were acquired from Thermo Fisher Scientific (Waltham, MA, USA). All other analytical-grade chemicals and reagents were sourced from commercial suppliers.

### 4.2. Preparation of WJD

A total of 1.67 g each of JDG, CX, BS, RG, MDP, and CEZ were weighed, and 3.33 g each of RS, CGC, and JNX were also weighed. All herbal materials were then pulverized into coarse granules (2–6 mm). Subsequently, 450 mL of distilled water was added, and the mixture was gently boiled down to 240 mL. Upon cooling, the concentrate was filtered through a 200-mesh sieve to yield the WJD extract.

The prepared WJD was concentrated under vacuum at 60 °C with a vacuum degree of −0.07 MPa to 40 mL, resulting in a crude drug concentration of 0.5 g/mL. A total of 600 mL of compact WJD solution was obtained, subpackaged, and stored at −20 °C for rat intragastric administration. Before intragastric administration to rats, representative constituents of the prepared WJD were quantified for quality control (Supplementary Table S1 and Figure S1).

### 4.3. Animals and Drug Administration

Specific pathogen-free (SPF) grade SD female rats (3 months old; unmated and non-pregnant, 240 ± 20 g; *n* = 48) were obtained from Liaoning Changsheng Biotechnology Co., Ltd. (Liaoning, China) (License No. SCXK(Liao)2020-0001). All procedures were reviewed and approved by the Animal Ethics Committee of Changchun University of Chinese Medicine (Approval No. 2024523). Experiments adhered to the Guidelines for the Care and Use of Laboratory Animals (National Research Council, 1996).

After a 7-day acclimatization period (temperature 21–25 °C, 35–65% humidity, 12-h light–dark cycle), the vaginal smear method was applied to monitor the estrous cycle of all rats during acclimation. Rats with regular cycles were stratified by estrous stage and body weight, then evenly randomized into six groups (*n* = 8/group): control group (CG; saline), model group (MG; saline), and Tongjingbao granule group (PG; according to the dosage specified in the instructions, the administration dose was determined to be 1.7 g/kg after clinical equivalent conversion, and the drug was dissolved in hot water for administration.). The daily clinical dosage of crude drugs in WD for adults is 60 g (calculated according to the standard body weight of an adult, which is 70 kg). Based on the conversion coefficient of body surface area between humans and rats of 6.3, the basic equivalent dosage for rats is 5.4 g/kg. In this study, crude drug dosages of 2.5 g/kg, 5 g/kg, and 10 g/kg were set as the low-, medium-, and high-dose groups, respectively, and the concentrated WJD was administered at 5 mL/kg, 10 mL/kg, and 20 mL/kg, respectively. These groups were designated as the low-dose WJD group (L-WJD; 5 mL/kg/day WJD), medium-dose WJD group (M-WJD; 10 mL/kg/day WJD), and high-dose WJD group (H-WJD; 20 mL/kg/day WJD). All groups except the CG were immersed in ice water at 0–4 °C at 9:00 every morning. The hind limbs and lower abdomen were completely submerged and the animals were closely monitored throughout the exposure. Ice-water exposure was immediately terminated when lethargy and hindlimb stiffness were observed, which served as the predefined criteria for discontinuing the procedure (immersion duration: approximately 10–15 min). After ice-water intervention, the rats were dried with towels and allowed to recover at room temperature. Recovery was monitored until normal activity and body temperature were restored, after which the animals were returned to their cages. Except for the CG, rats received daily subcutaneous injections of estradiol benzoate at 11:00 (0.4 mL/rat on days 1 and 12, and 0.2 mL/rat on days 2–11). From day 6, drugs were administered intragastrically at 12:00 once daily for 7 consecutive days. The CG and MG received an equivalent gavage volume of normal saline (10 mL/kg/day). On day 12, 1 h after intragastric administration, rats in the CG were intraperitoneally injected with normal saline, whereas all other groups were administered an intraperitoneal injection of oxytocin (0.2 mL/rat) to induce uterine contraction.

### 4.4. Sample Collection and Preparation

At 21:00 on day 12, all rats were kept fasting but allowed free access to water. On day 13, the final intragastric administration was performed at 9:00. One hour later (10:00), rats were anesthetized with an intraperitoneal injection of urethane (4.5 mL/kg). Following the induction of a stable surgical plane of anesthesia, blood samples were promptly withdrawn via the abdominal aorta. No animal was allowed to recover from anesthesia throughout the experiment. One milliliter of blood was transferred to a heparinized tube, thoroughly mixed, and hemorheological parameters were measured using an automated blood rheometer (Zibo Hengtuo Analytical Instrument Co., Ltd., Zibo, China). The remaining whole blood was placed in non-anticoagulant tubes at 25 °C for 30 min, then centrifuged at 3500 rpm for 15 min at 4 °C to separate serum. Serum samples were stored at −80 °C for subsequent analysis of biochemical indicators, blood components, and endogenous metabolites. Uterine tissues were dissected under clean conditions, washed with chilled normal saline, and divided according to the subsequent analyses. Samples for histopathological examination were fixed in 4% paraformaldehyde, whereas samples for Western blotting and RT-qPCR were snap-frozen in liquid nitrogen and stored at −80 °C until analysis.

Samples were randomly selected from the eight animals in each group according to the sample requirements of each assay. Samples from six rats per group were used for metabolomics and serum pharmacochemistry analyses, whereas uterine tissues from three individual rats per group were used for Western blotting and RT-qPCR. All reported n values represent independent biological replicates.

For serum pharmacochemistry and untargeted metabolomic profiling, 50 μL of serum was transferred into a 2-mL polypropylene centrifuge tube. Subsequently, 200 μL of ice-cold methanol–acetonitrile (1:1, *v*/*v*) was introduced, and the mixture was vortexed for 30 s. Samples were then incubated at −20 °C for 30 min before centrifugation at 12,000× *g* and 4 °C for 10 min. The 200-μL supernatant was harvested, lyophilized in vacuo, and reconstituted in 150 μL of 50% methanol. Following a second 30-s vortex, the solution underwent another centrifugation step (12,000× *g*, 4 °C, 10 min). Finally, the clarified supernatant was filtered through a 0.22-μm membrane syringe filter prior to instrumental analysis.

### 4.5. Observation Indices and Methods

#### 4.5.1. Observation of Body Weight, Writhing Response, and Organ Index

Body weight dynamics were monitored throughout the study across all experimental groups. Writhing responses were assessed for 30 min post-intraperitoneal oxytocin administration. The time elapsed until the initial writhing episode (i.e., from injection to first contraction) and the total count of writhing events during the observation window were quantified. The percentage inhibition of writhing for each group was subsequently calculated using the following formula:

(model group mean writhing response − treatment group mean writhing response)/Model group mean writhing response × 100%.

After excision, uterine tissues from all groups were collected, and adherent adipose tissue was carefully removed. The samples were rinsed with ice-cold physiological saline to eliminate blood contamination, blotted with filter paper to remove surface moisture, and subsequently weighed.

Uterine organ index = uterine weight/body weight × 100%.

#### 4.5.2. Hemorheological Analysis

Whole-blood and plasma viscosity (at low, medium, and high shear rates) were measured using an automated HT-100A blood rheometer (Zibo Hengtuo Analytical Instrument Co., Ltd., Zibo, China).

#### 4.5.3. Biochemical Indicators and Histopathological Analysis

Serum concentrations of TXB_2_, 6-keto-PGF_1_α, ET, IL-6, and TNF-α, along with uterine tissue levels of PGF_2_α and PGE_2_, were determined using rat-specific enzyme-linked immunosorbent assay (ELISA) kits. Uterine specimens were immersed in 4% paraformaldehyde for fixation, then subjected to conventional procedures including dehydration, clearing, paraffin impregnation, embedding, and microtome sectioning. The sections were then stained with hematoxylin and eosin (HE), dehydrated, and mounted for histological examination. Histomorphological features and pathological alterations were observed under an optical microscope (E100, Nikon Instruments Co., Ltd., Melville, NY, USA). All HE-stained uterine sections were assigned random codes to ensure blinded evaluation. An investigator blinded to group allocation randomly selected six high-power fields (400× magnification) from each section. The extent of stromal inflammatory infiltration and tissue edema was graded on a scale of 0–3: 0 (no change), 1 (mild), 2 (moderate), and 3 (severe). For each sample, the average score of the six fields was calculated for each parameter, and the results were subjected to statistical analysis across groups.

### 4.6. Serum Pharmacochemistry

#### 4.6.1. UPLC-Orbitrap Exploris 120 MS/Mass Spectrometry Analysis

Chromatographic separation was carried out using a reversed-phase Phenomenex Kinetex column (2.1 × 50 mm, 2.6 μm, 100 Å). Mobile phase A consisted of 0.01% acetic acid in water, while phase B was formulated as isopropanol–water (1:1, *v*/*v*). The gradient elution program comprised: 1% B for 0.5 min, a linear increase to 99% B over 6.5 min, held for 1 min, followed by a return to 1% B within 1 min and equilibration for 3 min. The column temperature was held at 25 °C, the flow rate maintained at 0.3 mL/min, and the injection volume set to 2 μL.

Mass spectrometric analysis was conducted using a heated ESI source operating in both positive and negative ion modes. Key parameters included: sheath gas flow at 50 arb, auxiliary gas at 15 arb, capillary temperature of 320 °C, full MS resolution of 60,000, MS/MS resolution of 15,000, SNCE levels of 20/30/40, and spray voltages of 3.8 kV (POS) and −3.4 kV (NEG).

#### 4.6.2. Data Analysis and Compound Identification

Raw data were acquired using a UPLC-Orbitrap Exploris 120 MS system and processed with Xcalibur software (v4.4, Thermo Fisher Scientific). The serum pharmacochemistry components of WJD were identified based on the self-built compound database of Personalbio Technology Co., Ltd. (Shanghai, China). The database integrated compound information, including compound name, molecular formula, molecular weight, chemical structure, and characteristic product ion fragments. The acquired MS/MS spectra were matched against the in-house database, and compound identification was further supported by comparison with public databases, including ChemSpider, PubChem, ChemBank, MassBank, and Elsevier ScienceDirect. Based on accurate molecular mass, fragment ion information, and database matching results, the potential prototype components and metabolites of WJD translocated into serum were identified.

### 4.7. Network Pharmacology

Potential bioactive compounds derived from serum pharmacochemistry were chosen as the basis for subsequent network pharmacology analysis. To ensure clinical relevance, all database queries were restricted to Homo sapiens. The predicted targets of these compounds were identified utilizing SwissTargetPrediction (http://www.swisstargetprediction.ch/, accessed on 12 May 2026), applying a probability threshold >0.5. Following data integration and duplicate removal, a target library specific to WJD components was established. Concurrently, disease-related targets were gathered from GeneCards (http://www.genecards.org/, accessed on 12 May 2026) and DrugBank (https://www.drugbank.ca/, accessed on 12 May 2026) by searching the keyword “primary dysmenorrhea”. Targets from GeneCards were filtered based on a relevance score ≥10, while DrugBank entries underwent manual verification. After merging, deduplication, and manual screening, only biologically relevant targets were retained. The intersection between WJD component-related targets and primary dysmenorrhea-associated targets was determined using Venny 2.1.0. These overlapping targets were then imported into STRING (https://cn.string-db.org/, accessed on 12 May 2026) with the species set to Homo sapiens and a minimum interaction score of 0.4, after which disconnected nodes were hidden. The protein–protein interaction (PPI) network was assembled and visualized via Cytoscape (version 3.10.3). Network topology analysis, including Degree and Eigenvector Centrality, was conducted using the CytoNCA plugin; nodes surpassing the median threshold were classified as core targets. Functional enrichment analysis, including Gene Ontology (GO) terms and Kyoto Encyclopedia of Genes and Genomes (KEGG) pathways, was executed through the DAVID database (https://davidbioinformatics.nih.gov/, accessed on 12 May 2026), setting statistical significance at FDR < 0.05. The top 30 significantly enriched GO terms and KEGG pathways were graphically represented using the Bioinformatics Platform (https://www.bioinformatics.com.cn/, accessed on 12 May 2026). Ultimately, a component-target-pathway (C-T-P) interactive network was constructed in Cytoscape, integrating associations among compounds, putative targets, and signaling pathways. Highly connected nodes and targets were prioritized based on network degree for subsequent molecular docking studies. For cross-species comparison, human candidate targets selected for experimental evaluation were cross-referenced with their corresponding Rattus norvegicus orthologues using the Ensembl database.

### 4.8. Molecular Docking

The structural data for the WJD serum components were retrieved from PubChem (https://pubchem.ncbi.nlm.nih.gov/, accessed on 13 May 2026). Prior to docking, ligand structures underwent energy minimization using Chem3D (MM2 force field, convergence criterion of 0.01) and were subsequently converted into PDB format. Crystal structures of the target proteins (EGFR 8A27, AKT1 8UW7, ESR1 8ARR, MMP9 8K5Y, MAPK3 6GES, TNF 8Z8M) were acquired from the RCSB PDB (https://www.rcsb.org/, accessed on 13 May 2026). The native ligand-binding pocket was selected as the docking site. Protein preparation was executed in PyMOL to eliminate water molecules, bound ligands, and heteroatoms. AutoDockTools 1.5.7 was utilized to append hydrogen atoms, compute Gasteiger charges, and generate PDBQT files. A grid box was constructed to encapsulate the original binding pocket, and molecular docking was conducted using AutoDock Vina. The resulting conformations were prioritized based on binding energy, with the optimal pose subsequently visualized using PyMOL 3.1.1.

### 4.9. Untargeted Serum Metabolomics

#### 4.9.1. Sample Selection and Study Design

Based on the pharmacodynamic results, the H-WJD group, which showed the most pronounced pharmacological effects, was selected for untargeted serum metabolomics analysis. This analysis was performed as an exploratory approach to identify potential metabolic alterations associated with WJD treatment. Since only the H-WJD group was included for metabolomics profiling, this design was not intended to evaluate dose-dependent metabolic effects, but rather to provide preliminary screening of potential differential metabolites.

#### 4.9.2. UPLC and Mass Spectrometry Conditions

Chromatographic separation was achieved on an ACQUITY UPLC HSS T3 column (2.1 × 100 mm, 1.8 μm, 100 Å) under the following conditions: flow rate, 0.4 mL/min; column temperature, 40 °C; autosampler temperature, 8 °C; and injection volume, 2 μL. Mobile phases comprised water with 0.1% formic acid (A) and acetonitrile (B). The gradient profile was programmed as follows: 5% B (0–1 min), ramp to 95% B (1–4.7 min), hold at 95% B (4.7–6 min), and re-equilibrate to 5% B (6.1–8.5 min).

Mass spectrometric analysis was performed using a Thermo Orbitrap Exploris 120 system controlled by Xcalibur software (v. 4.7). Data-dependent acquisition (DDA) was employed in both positive and negative ion modes via a heated electrospray ionization (HESI) source. Source parameters were: spray voltage, 3.5 kV (POS) and −3.0 kV (NEG); sheath gas, 40 arbs; auxiliary gas, 10 arbs; capillary temperature, 320 °C; and auxiliary gas heater, 300 °C. Full-scan MS1 spectra (*m*/*z* 70–1000) were acquired at 60,000 resolutions with standard AGC and 100 ms Max IT. The top 4 most abundant precursors were selected for MS/MS fragmentation using dynamic exclusion (4 s). MS2 spectra were recorded at 15,000 resolutions with HCD at 30% collision energy, standard AGC, and automatic Max IT.

#### 4.9.3. Data Processing and Quality Control

Raw MS data were analyzed using MS-DIAL (v. 4.9.221218), which performs peak detection, retention time alignment, noise filtering, and metabolite annotation. Features identified in <50% of QC samples were excluded. Missing values were imputed via the software’s built-in gap-filling algorithm, and the final dataset was normalized to total ion current (TIC). Only metabolites showing RSD <30% across QC samples were retained for subsequent statistical analysis.

#### 4.9.4. Metabolite Annotation and Identification Criteria

Metabolite annotation was performed based on the in-house PSNGM database developed by Personalbio Technology Co., Ltd., which integrates a self-built standard database and multiple public databases, including mzCloud, LIPID MAPS, HMDB, MoNA, and NIST 2020 MS/MS. Metabolite annotation confidence was assigned according to the criteria proposed by the Metabolomics Standards Initiative. Level 1 metabolites were confirmed by comparison with authentic standards, including matched accurate mass, retention time, and MS/MS fragmentation spectra. Level 2 metabolites were putatively annotated without reference standards, based on accurate mass, isotopic distribution, MS/MS fragmentation patterns, and comparison with spectra in public databases.

Metabolite structural annotation was achieved through multidimensional matching of retention time, molecular mass within ±10 ppm, MS/MS spectra, and characteristic fragmentation patterns retrieved from databases. All annotated metabolites were manually reviewed. Only metabolites annotated at MSI Level 2 or above were retained for downstream analysis.

#### 4.9.5. Multivariate Statistical Analysis and Pathway Enrichment

Following preprocessing, the serum metabolomic dataset was subjected to multivariate modeling via principal component analysis (PCA) and orthogonal partial least-squares discriminant analysis (OPLS-DA). Univariate two-group comparison was conducted using Student’s *t*-test to obtain nominal *p*-values. The Benjamini–Hochberg procedure was applied for multiple-testing correction to generate FDR-adjusted *p*-values. Metabolites with FDR-adjusted *p* < 0.05 were regarded as statistically significant. Candidate differential metabolites were screened based on variable importance in projection (VIP) ≥ 1.0, fold change (FC) ≥ 1.2 or ≤ 0.83, and nominal *p* < 0.05, whereas candidates meeting these criteria but with FDR-adjusted *p* ≥ 0.05 were treated as exploratory candidates. The candidate metabolites were subsequently subjected to exploratory pathway enrichment analysis using MetaboAnalyst 5.0 against the Rattus norvegicus metabolic network.

### 4.10. Convergence of Network Pharmacology and Metabolomics Insights

A “pathway–metabolite–target–compound” network was established using the Metscape plugin in Cytoscape 3.10.3, integrating findings from metabolomics and network pharmacology analyses.

### 4.11. Western Blot Analysis of Uterine Tissue

Uterine tissues were homogenized in ice-cold RIPA buffer (P0013B, Beyotime Biotechnology, Shanghai, China) containing a protease and phosphatase inhibitor cocktail (ST506, Beyotime Biotechnology). After centrifugation at 12,000× *g* for 15 min at 4 °C, the supernatants were collected. Protein concentrations were measured using a BCA Protein Assay Kit (P0012, Beyotime Biotechnology). Equal amounts of protein from each sample were resolved by SDS-PAGE and subsequently transferred onto PVDF membranes. The membranes were blocked with 5% bovine serum albumin (BSA) at room temperature for 1 h, followed by an overnight incubation at 4 °C with the following primary antibodies: phospho-AKT (1:1000, #4060), AKT (1:2000, #9272), phospho-ERK1/2 (1:2000, #4370), ERK1/2 (1:1000, #4695), phospho-NF-κB p65 (1:1000, #3033), NF-κB p65 (1:1000, #8242), COX-2 (1:1000, #12282) (all from Cell Signaling Technology, Danvers, MA, USA), and GAPDH (1:5000, #T00004, Affinity Biosciences, Cincinnati, OH, USA).

Following three washes with TBST, the membranes were incubated with the appropriate horseradish peroxidase (HRP)-conjugated secondary antibodies at room temperature for 1 h. Protein bands were visualized using an enhanced chemiluminescence (ECL) detection kit (BL520B, Biosharp) and acquired with a WB-one Pro fluorescence and chemiluminescence imaging system (Servicebio, Wuhan, China). Band intensities were quantified using ImageJ1.53a software. The levels of phosphorylated proteins were normalized to their corresponding total protein levels (p-AKT/AKT, p-ERK1/2/ERK1/2, and p-NF-κB/NF-κB), whereas COX-2 expression was normalized to GAPDH.

### 4.12. Real-Time Quantitative Polymerase Chain Reaction (RT-qPCR)

Total RNA was extracted using an RNA isolation solution (Wuhan Saiver Biotechnology Co., Ltd., Wuhan, China; catalog no. G3013). The concentration and purity of the extracted RNA were assessed with a NanoDrop 2000 spectrophotometer (Thermo Fisher Scientific, Wilmington, DE, USA) and adjusted to 200 ng/μL. First-strand cDNA was synthesized using 5× SweScript All-in-One SuperMix (catalog no. G3337; Wuhan Saiver Biotechnology Co., Ltd.). Quantitative real-time PCR (RT-qPCR) was performed in triplicate with 2× Universal Blue SYBR Green qPCR Master Mix (catalog no. G3326; Wuhan Saiver Biotechnology Co., Ltd.). The thermal cycling protocol comprised an initial denaturation at 95 °C for 30 s, followed by 40 cycles of denaturation at 95 °C for 15 s and annealing/extension at 60 °C for 30 s, and concluded with melting curve analysis. Glyceraldehyde-3-phosphate dehydrogenase (GAPDH) was used as the housekeeping gene, and relative gene expression was calculated using the 2^−ΔΔCt^ method. Primer sequences are listed in Table 2.

### 4.13. Statistical Analysis

Statistical analyses were performed using SPSS version 26.0 (IBM Corp., Armonk, NY, USA). Data are presented as mean ± standard deviation (SD). Normality of distribution was assessed via the Shapiro–Wilk test, and homogeneity of variance was evaluated using Levene’s test. For multi-group comparisons, one-way analysis of variance (ANOVA) was applied, followed by Tukey’s multiple comparison test. When comparing two groups, Student’s *t*-test was employed. Repeated measurements, such as body weight changes over time, were analyzed using two-way repeated-measures ANOVA to evaluate the effects of treatment, time, and their interaction. A *p*-value below 0.05 was considered statistically significant, while *p* < 0.01 was interpreted as highly significant. Graphs were generated using GraphPad Prism 10 (GraphPad Software, San Diego, CA, USA).

All assessments, including the writhing behavior test, histopathological examination, ELISA, qPCR, and metabolomics analysis, were performed in a blinded fashion.

## 5. Conclusions

In this study, the pharmacological effects of WJD on cold coagulation and blood stasis syndrome-associated primary dysmenorrhea (CCBS-PD) were evaluated by integrating pharmacodynamic assessment with serum pharmacochemistry, network pharmacology, and metabolomics analyses. The results suggest that multiple serum-exposed constituents of WJD might be linked to the modulation of inflammatory signaling pathways, including PI3K/AKT, MAPK, and NF-κB, potentially through targets such as EGFR, AKT1, MAPK3, and TNF. Exploratory metabolomics analysis further suggested that WJD intervention may be associated with changes in amino acid-related metabolism, accompanied by improvements in prostaglandin-related indicators and inflammatory responses. Collectively, these findings provide supportive evidence for the multi-component and multi-target pharmacological effects of WJD in CCBS-PD and offer a basis for further investigation of its pharmacological actions. Nevertheless, the specific molecular mechanisms and key bioactive constituents remain to be clarified through targeted metabolomics and functional validation studies.

## Figures and Tables

**Table 1:** Hemorheology results (*n* = 8).

Group	L-WBV 10 s^−1^ (mPa·s)	M-WBV 60 s^−1^ (mPa·s)	H-WBV 150 s^−1^ (mPa·s)	PV (mPa·s)
CG	12.22 ± 1.32	6.79 ± 0.80	4.96 ± 0.98	1.65 ± 0.34
MG	20.64 ± 1.44 ^###^	9.75 ± 0.97 ^###^	7.19 ± 0.49 ^###^	2.11 ± 0.15 ^###^
PG	13.52 ± 0.96 ***	7.35 ± 0.63 ***	5.50 ± 0.52 ***	1.71 ± 0.05 ***
L-WJD	13.58 ± 0.79 ***	7.37 ± 0.66 ***	5.49 ± 0.41 ***	1.81 ± 0.15 **
M-WJD	13.62 ± 1.14 ***	7.32 ± 0.59 ***	5.45 ± 0.50 ***	1.78 ± 0.06 ***
H-WJD	12.91 ± 1.02 ***	7.19 ± 0.19 ***	5.40 ± 0.31 ***	1.75 ± 0.06 ***

^###^ *p* < 0.001 vs. CG; ** *p* < 0.01, *** *p* < 0.001 vs. MG.

**Table 2:** Sequence of primers used for the RT-qPCR analysis.

Gene	Forward Primer (5′-3′)	Reverse Primer (5′-3′)
AKT1	5′-CTGGAGGACAACGACTATGGC-3′	5′-AGCCTCTGTGTAGGGTCCTTCTT-3′
EGFR	5′-AGAACAACACCCTGGTCTGGAA-3′	5′-CCACCACTACTATGAAGAGAGGC-3′
MAPK3	5′-GAGACATCCTCAGAGCACCCA-3′	5′-TGTTGATAAGCAGATTGGAGGG-3′
TNFα	5′-CCACCACGCTCTTCTGTCTACTG-3′	5′-TGGGCTACGGGCTTGTCACT-3′
ESR1	5′-GCCAAGGAGACTCGCTACTGT-3′	5′-CTGACGCTTGTGCTTCAACAT-3′
MMP9	5′-GCCGACTTATGTGGTCTTCCC-3′	5′-GCCCGAGTGTAACCATAGCG-3′
CASP3	5′-GAAAGCCGAAACTCTTCATCAT-3′	5′-ATGCCATATCATCGTCAGTTCC-3′

## Data Availability

The original contributions presented in the study are included in the article and Supplementary Materials. The raw metabolomics and serum pharmacochemistry data have been submitted to the MetaboLights database under accession numbers MTBLS14535 and MTBLS14534.

## Supplementary Materials

The following supporting information can be downloaded at: https://www.mdpi.com/article/10.3390/ph19091385/s1. Table S1. Contents of gallic acid, paeoniflorin, liquiritin, ferulic acid, cinnamic acid, cinnamaldehyde, paeonol and ammonium glycyrrhizinate in WJD. Figure S1. Chromatograms of content determination (A: Mixed reference substance chromatogram; 1: Gallic acid, 2: Paeoniflorin, 3: Liquiritin, 4: Ferulic acid, 5: Cinnamic acid, 6: Cinnamaldehyde, 7: Paeonol, 8: Ammonium glycyrrhizinate; B: Chromatogram of Wenjing Decoction sample). Table S2. Effects of WJD on writhing response of rats in each group (*n* = 8). Table S3. Uterine organ index data (*n* = 8). Table S4. Scores of interstitial inflammatory infiltrations and tissue edema in each group (*n* = 6). Table S5. Expression levels of related serum factors in rats of each group (*n* = 6). Table S6. The candidate differential metabolites in the serum of rats (Model vs. Control). Table S7. The candidate differential metabolites in the serum of rats (Model vs. H-WJD). Table S8. Metabolomic pathway enrichment data. Figure S2. Total ion chromatograms (TIC) of serum metabolites in Control, Model and WJD groups under positive and negative ion modes. Table S9. Identification of prototype components in WJD rats after oral administration of WJD. Table S10. Identification of metabolic components in WJD rats after oral administration of WJD. Table S11. Network pharmacology targets. Table S12. Topological analysis parameters. Table S13. Degree values of core compounds.

## Abbreviations

The following abbreviations are used in this manuscript:

WJD	Wenjing Decoction
CCBS-PD	Cold Coagulation and Blood Stasis Primary Dysmenorrhea
PD	Primary dysmenorrhea
NSAIDs	nonsteroidal anti-inflammatory drugs
SPF	Specific pathogen-free
CG	control group
MG	model group
PG	Tongjingbao granule group
L-WJD	low-dose WJD group
M-WJD	medium-dose WJD group
H-WJD	high-dose WJD group
TXB_2_	Thromboxane B_2_
6-keto-PGF_1_α	6-keto-prostaglandin F_1_α
ET	Endothelin
E_2_	Estradiol
IL-6	Interleukin-6
TNF-α	tumor necrosis factor-α
PGF_2_α	Prostaglandin F_2_α
PGE_2_	Prostaglandin E_2_
HE	hematoxylin/eosin
POS	positive ion mode
NEG	negative ion mode
QC	quality control
PCA	principal component analysis
OPLS-DA	orthogonal partial least squares discriminant analysis
VIP	variable importance in projection
FC	fold change
PPI	protein–protein interaction
GO	Gene Ontology
KEGG	Kyoto Encyclopedia of Genes and Genomes
C–T–P	component–target–pathway
PDB	Protein Data Bank
SD	standard deviation
ANOVA	one-way analysis of variance
TXA_2_	Thromboxane A_2_
PGI_2_	prostaglandin I_2_
RT-qPCR	reverse transcription quantitative real-time polymerase chain reaction
AKT	Protein kinase B
P-AKT	Phosphorylated protein kinase B
ERK1/2	Extracellular signal-regulated kinase 1/2
P-ERK1/2	Phosphorylated extracellular signal-regulated kinase 1/2
NF-κB	Nuclear factor kappa B
P-NF-κB	Phosphorylated nuclear factor kappa B
COX-2	Cyclooxygenase-2
GAPDH	Glyceraldehyde-3-phosphate dehydrogenase
