# Supplementary material for: Pharmacological Effects of Wenjing Decoction in a Rat Model of Cold Coagulation and Blood Stasis Primary Dysmenorrhea: An Integrated Analysis of Serum Pharmacochemistry, Network Pharmacology and Metabolomics
Source: Pharmaceuticals (Basel). 2026 Sep 1;19(9):1385. doi: 10.3390/ph19091385 (PMC13610681; doi:10.3390/ph19091385)
Supplement: Supplementary file 1 [file pharmaceuticals-19-01385-s001.zip › pharmaceuticals-4485007-supplementary.pdf]

Supplementary Material

1 Determination of gallic acid, paeoniflorin, liquiritin, ferulic acid, cinnamic acid, cinnamaldehyde, paeonol and ammonium glycyrrhizinate in Wenjing Decoction (WJD)

1.1 Preparation of mixed reference solution

Appropriate amounts of gallic acid, cinnamaldehyde, cinnamic acid, ferulic acid, paeoniflorin, paeonol, liquiritin and ammonium glycyrrhizinate were accurately weighed, dissolved with methanol and diluted to constant volume in a volumetric flask. After shaking evenly, the mixed reference solution was obtained with concentrations of 0.0445mg/mL, 0.0858mg/mL, 0.0310mg/mL, 0.0700mg/mL, 0.1062mg/mL, 0.0521mg/mL, 0.0810mg/mL and 0.1330 mg/mL, respectively.

1.2 Preparation of test sample

Take 1 mL of WJD extract (0.1 g/mL) was transferred into a 10 mL volumetric flask, and diluted to the scale mark with methanol to obtain the test sample solution.

1.3 Chromatographic conditions

The analysis was performed on an Agilent HC-C18 column (4.6 × 250 mm, 5 μm, 170 Å). Acetonitrile (A) - 0.1% phosphoric acid aqueous solution (B) was used as mobile phase with gradient elution: 0–5 min, 0% A → 3% A; 5–15 min, 3% A → 15% A; 15–20 min, 15% A; 20–25 min, 15% A → 21% A; 25–28 min, 21% A; 28–55 min, 21% A → 45% A; 55–75 min, 45% A → 70% A; 75–100 min, 70% A.The detection wavelength was set at 220 nm, column temperature was 30 °C, and flow rate was 0.8 mL/min. The injection volume of mixed reference solution and WJD sample solution was 10 μL.

1.4 Determination results of eight components in WJD

Table S1. Contents of gallic acid, paeoniflorin, liquiritin, ferulic acid, cinnamic acid, cinnamaldehyde, paeonol and ammonium glycyrrhizinate in WJD.

Analytes	Content of test sample（μg/g）	Average content（μg/g）
	77.38	
Gallic acid	71.45	76.35
	80.21	
Paeoniflorin	22.68	26.41

	25.49	
	31.06	
Liquiritin	6.71<br>8.04<br>5.33	6.69
Ferulic acid	47.21<br>53.19<br>50.56	50.32
Cinnamic acid	128.42<br>110.47<br>109.66	116.18
Cinnamaldehyde	110.71<br>102.40<br>129.63	114.25
Paeonol	226.41<br>217.57<br>232.38	225.45
Ammonium glycyrrhizinate	542.92<br>538.75<br>511.99	531.22

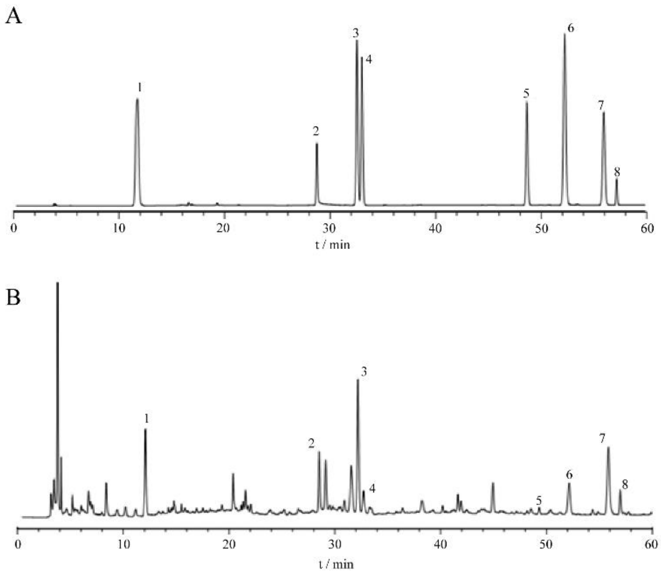

Figure S1. Chromatograms of content determination (A: Mixed reference substance chromatogram; 1: Gallic acid, 2: Paeoniflorin, 3: Liquiritin, 4: Ferulic acid, 5: Cinnamic acid, 6: Cinnamaldehyde, 7: Paeonol, 8: Ammonium glycyrrhizinate; B: Chromatogram of Wenjing Decoction sample).

2 Writhing response

Table S2. Effects of WJD on writhing response of rats in each group (n=8).

Group	Writhing latency (min)	Writhing times within 30 minutes	Writhing inhibition rate (%)
CG	/	/	/
MG	3.63±1.06###	29.75±3.77###	/
PG	4.88±1.46**	12.13±3.81***	59.23
L-WJD	4.37±0.74***	22.38±2.13***	24.77
M-WJD	8.38±1.19***	15.88±1.73***	48.30
H-WJD	10.88±1.46***	11.25±3.81***	62.18

Note: #p < 0.05, ##p < 0.01, ###p < 0.001 *vs.* CG; *p < 0.05, **p < 0.01, ***p < 0.001 *vs.* MG.

3 Uterine organ index

Table S3. Uterine organ index data (n=8).

Group	Final body weight (g)	Uterine wet weight (g)	Uterine index (%)
CG	257.91±6.70	0.87±0.22	0.34±0.09
MG	205.86±13.69	1.36±0.36	0.66±0.18$^{###}$
PG	210.78±8.79	1.09±0.27	0.52±0.14
L-WJD	211.65±10.16	1.12±0.32	0.53±0.16
M-WJD	203.95±12.66	1.09±0.27	0.52±0.11
H-WJD	206.97±20.37	0.97±0.07	0.47±0.04**

Note: #*p* < 0.05, ##*p* < 0.01, ###*p* < 0.001 *vs.* CG; *p < 0.05, **p < 0.01, ***p < 0.001 *vs.* MG.

4 Serum Biochemistry and Histopathological Analysis

Table S4. Scores of interstitial inflammatory infiltrations and tissue edema in each group (n=6).

Group	Inflammation score	Edema score
CG	0.33±0.52	0.50±0.55
MG	2.67±0.52$^{###}$	2.83±0.41$^{###}$
PG	1.50±0.55**	1.83±0.41**
L-WJD	2.50±0.55	2.67±0.52
M-WJD	1.83±0.41*	1.67±0.52**
H-WJD	1.16±0.41***	1.00±0.63***

Note: #*p* < 0.05, ##*p* < 0.01, ###*p* < 0.001 *vs.* CG; *p < 0.05, **p < 0.01, ***p < 0.001 *vs.* MG.

Table S5. Expression levels of related serum factors in rats of each group (n=6).

Group	TXB_2 (pg/mL)	6-keto-$PGF_{1\alpha}$ (pg/mL)	ET (μg/mL)	IL-6 (pg/mL)	TNF-α (pg/mL)	$PGF_{2\alpha}$ (ng/mL)	PGE_2 (ng/mL)
CG	2.08±0.33	0.96±0.10	1.46±0.29	0.78±0.11	1.42±0.19	0.12±0.02	2.90±0.55
MG	2.68±0.18$^{\#\#}$	0.67±0.07$^{\#\#\#}$	3.04±0.52$^{\#\#\#}$	1.30±0.06$^{\#\#\#}$	2.04±0.33$^{\#\#}$	0.27±0.04$^{\#\#\#}$	2.01±0.46$^{\#}$
PG	2.11±0.32**	0.77±0.07*	2.10±0.38**	1.08±0.10***	1.41±0.32**	0.14±0.03***	2.56±0.36*
L-WJD	2.14±0.22***	0.87±0.12**	2.10±0.47**	1.07±0.08***	1.58±0.26*	0.19±0.02**	2.43±0.23
M-WJD	2.04±0.10***	0.85±0.11**	1.98±0.40**	1.10±0.11**	1.14±0.07***	0.17±0.02***	2.75±0.41*
H-WJD	2.08±0.19***	0.92±0.11***	1.47±0.29***	0.85±0.12***	1.10±0.16***	0.13±0.02***	2.76±0.34**

Note: #$p < 0.05$, ##$p < 0.01$, ###$p < 0.001$ *vs.* CG; *$p < 0.05$, **$p < 0.01$, ***$p < 0.001$ *vs.* MG.

5 Metabolomics Results

Table S6. The candidate differential metabolites in the serum of rats (Model *vs.* Control).

NO.	Name	Formula	t(min)	Measured ion (m/z)	Ion mode	FC	VIP	Nominal. *p*	FDR-adjusted *p*	Regulation	Level
S1	PI (18:3/TXB$_2$)	$C_{47}H_{79}O_{17}P$	3.376	945.49133	$[M-H]^-$	1.31	1.45	0.0486	0.3242	Up	II
S2	3-Indoxyl sulphate	$C_8H_7NO_4S$	3.687	212.00266	$[M-H]^-$	2.07	1.52	0.0414	0.3027	Up	I
S3	3, 5-Tetradecadiencarnitine	$C_{21}H_{37}NO_4$	4.654	368.28241	$[M+H]^+$	1.53	1.59	0.0453	0.3800	Up	II
S4	Prolyl-Threonine	$C_9H_{16}N_2O_4$	1.155	217.12012	$[M+H]^+$	2.50	1.61	0.0472	0.3856	Up	I
S5	Isohyodeoxycholic acid	$C_{24}H_{40}O_4$	5.073	375.29227	$[M+H-H_2O]^+$	5.12	1.63	0.0409	0.3671	Up	II
S6	p-Cresol glucuronide	$C_{13}H_{16}O_7$	3.690	283.08276	$[M-H]^-$	2.16	1.65	0.0243	0.2424	Up	II
S7	Taurodeoxycholic Acid	$C_{26}H_{45}NO_6S$	5.605	500.30771	$[M+H]^+$	2.87	1.68	0.0400	0.3633	Up	II
S8	L-Isoleucyl-L-alanine	$C_9H_{18}N_2O_3$	1.114	203.14079	$[M+H]^+$	1.68	1.75	0.0231	0.3002	Up	II
S9	p-Cresol	C_7H_8O	4.000	107.05078	$[M-H]^-$	3.44	1.80	0.0114	0.1729	Up	I

S10	3-Hydroxy-4-aminopyridine sulfate	$C_5H_6N_2O_4S$	4.000	189.00336	$[M-H]^-$	3.46	1.80	0.0116	0.1737	Up	II
S11	LysoPC(22:5/0:0)	$C_{30}H_{52}NO_7P$	5.633	570.35944	$[M+H]^+$	1.57	1.81	0.0131	0.2382	Up	II
S12	N2-Acetylornithine	$C_7H_{14}N_2O_3$	0.676	173.09351	$[M-H]^-$	2.90	1.82	0.0188	0.2196	Up	II
S13	Chenodeoxycholic Acid	$C_{24}H_{40}O_4$	4.987	437.29153	$[M-HCOO]^-$	4.38	1.83	0.0072	0.1393	Up	II
S14	Sulfolithocholylglycine	$C_{26}H_{43}NO_7S$	4.303	512.26941	$[M-H]^-$	5.34	1.84	0.0083	0.1493	Up	II
S15	Murocholic acid	$C_{24}H_{40}O_4$	4.987	391.28601	$[M-H]^-$	4.59	1.87	0.0055	0.1224	Up	II
S16	LysoPC(18:3/0:0)	$C_{26}H_{48}NO_7P$	5.331	518.328	$[M+H]^+$	1.50	1.90	0.0147	0.2482	Up	II
S17	Ursodiol	$C_{24}H_{40}O_4$	4.984	357.28137	$[M+H-2H_2O]^+$	4.77	1.91	0.0071	0.1827	Up	II
S18	Pseudouridine	$C_9H_{12}N_2O_6$	0.938	243.06279	$[M-H]^-$	1.32	1.91	0.0080	0.1473	Up	I
S19	Taurocholic acid	$C_{26}H_{45}NO_7S$	4.664	514.28522	$[M-H]^-$	7.40	1.94	0.0033	0.0966	Up	I
S20	Dihydrodaidzein 7-O-glucuronide	$C_{21}H_{20}O_{10}$	3.874	431.09937	$[M-H]^-$	1.38	1.95	0.0037	0.1012	Up	II
S21	Epiandrosterone sulfate	$C_{19}H_{30}O_5S$	6.354	369.17453	$[M-H]^-$	1.72	1.95	0.0037	0.1012	Up	I

S22	1,4-Butanediamine	$C_4H_{12}N_2$	7.407	89.00907	$[M+H]^+$	1.23	2.03	0.0032	0.1328	Up	I
S23	Glycodeoxycholic acid	$C_{26}H_{43}NO_5$	5.041	448.30737	$[M-H]^-$	5.76	2.03	0.0035	0.0997	Up	I
S24	Tauroursodeoxycholic acid	$C_{26}H_{45}NO_6S$	5.457	498.29016	$[M-H]^-$	6.91	2.07	0.0024	0.0834	Up	I
S25	Aldosterone	$C_{21}H_{28}O_5$	7.366	359.19055	$[M-H]^-$	16.07	2.10	0.0007	0.0603	Up	I
S26	Taurolithocholic acid	$C_{26}H_{45}NO_5S$	4.502	482.29526	$[M-H]^-$	11.89	2.11	0.0007	0.0594	Up	I
S27	Glycocholate	$C_{26}H_{43}NO_6$	4.545	464.30212	$[M-H]^-$	10.51	2.12	0.0013	0.0675	Up	I
S28	Deoxycholic acid 3-glucuronide	$C_{30}H_{48}O_{10}$	4.781	567.31866	$[M-H]^-$	83.04	2.34	0.0000	0.0105	Up	II
S29	4-Vinylphenol sulfate	$C_8H_8O_4S$	4.319	199.00754	$[M-H]^-$	21.66	2.48	0.0000	0.0024	Up	II
S30	Thromboxane B2	$C_{20}H_{34}O_6$	4.400	369.22882	$[M-H]^-$	0.55	1.49	0.0496	0.3278	Down	I
S31	Oxalic acid	$C_2H_2O_4$	0.504	88.9882	$[M-H]^-$	0.67	1.49	0.0390	0.2955	Down	I
S32	Allantoin	$C_4H_6N_4O_3$	0.671	157.03709	$[M-H]^-$	0.80	1.54	0.0342	0.2792	Down	I
S33	4-Pyridoxic acid	$C_8H_9NO_4$	1.181	182.04633	$[M-H]^-$	0.39	1.54	0.0343	0.2792	Down	I

S34	4-Hydroxybutyric acid	$C_4H_8O_3$	1.183	103.0403	$[M-H]^-$	0.30	1.55	0.0460	0.3158	Down	I
S35	L-Threonic acid	$C_4H_8O_5$	0.665	135.03026	$[M-H]^-$	0.75	1.56	0.0318	0.2675	Down	I
S36	Maleic acid	$C_4H_4O_4$	1.098	115.00406	$[M-H]^-$	0.48	1.58	0.0473	0.3200	Down	I
S37	9,10-Dhome	$C_{18}H_{34}O_4$	5.261	349.21567	$[M-Cl]^-$	0.53	1.62	0.0322	0.2693	Down	II
S38	Linolenic acid	$C_{18}H_{30}O_2$	5.274	279.23413	$[M-H]^+$	0.59	1.63	0.0299	0.3260	Down	I
S39	L-Kynurenine	$C_{10}H_{12}N_2O_3$	1.699	209.09377	$[M+H]^+$	0.66	1.63	0.0285	0.3238	Down	I
S40	Prostaglandin B1	$C_{20}H_{32}O_4$	5.276	337.23755	$[M+H]^+$	0.58	1.63	0.0301	0.3261	Down	I
S41	m-Coumaric acid	$C_9H_8O_3$	1.081	165.05583	$[M-H]^+$	0.63	1.63	0.0343	0.3431	Down	I
S42	3-Hydroxybutyric acid	$C_4H_8O_3$	1.129	103.04028	$[M-H]^-$	0.41	1.64	0.0234	0.2384	Down	I
S43	LysoPC(P-18:0)	$C_{26}H_{54}NO_6P$	6.329	530.35168	$[M+Na]^+$	0.42	1.65	0.0285	0.3238	Down	II
S44	9-Oxo-ODE	$C_{18}H_{30}O_3$	5.821	295.22919	$[M+H]^+$	0.59	1.65	0.0294	0.3254	Down	II
S45	N-Acetyl-L-methionine	$C_7H_{13}NO_3S$	3.230	190.05476	$[M-H]^-$	0.63	1.68	0.0165	0.2058	Down	II
S46	Diethyl decanedioate	$C_{14}H_{26}O_4$	4.577	257.17639	$[M-H]^-$	0.59	1.71	0.0148	0.1965	Down	II

S47	3-Indole carboxylic acid glucuronide	$C_{15}H_{15}NO_8$	3.341	336.07333	$[M-H]^-$	0.36	1.71	0.0162	0.2039	Down	II
S48	α-ketoadipic acid	$C_6H_8O_5$	2.065	115.04037	$[M-H-CO_2]^-$	0.61	1.77	0.0174	0.2120	Down	II
S49	β-Guanidinopropionic acid	$C_4H_9N_3O_2$	0.651	130.06255	$[M-H]^-$	0.73	1.78	0.0148	0.1966	Down	I
S50	N-Isobutyrylglycine	$C_6H_{11}NO_3$	2.009	144.06700	$[M-H]^-$	0.49	1.81	0.0102	0.1636	Down	I
S51	Methyl palmitate	$C_{17}H_{34}O_2$	4.664	271.29166	$[M+H]^+$	0.27	1.86	0.0236	0.3025	Down	I
S52	Oleoyl ethanolamide	$C_{20}H_{39}NO_2$	4.682	326.47851	$[M+H]^+$	0.27	1.86	0.0244	0.3066	Down	II
S53	Prostaglandin $F_{2\alpha}$	$C_{20}H_{34}O_5$	4.503	353.23407	$[M-H]^-$	0.37	1.93	0.0052	0.1186	Down	I
S54	Creatinine	$C_4H_7N_3O$	0.630	114.06709	$[M-H]^+$	0.76	1.93	0.0063	0.1733	Down	I
S55	Citric acid	$C_6H_8O_7$	0.954	191.02016	$[M-H]^-$	0.47	1.93	0.0076	0.1440	Down	I
S56	L-Ascorbic acid 2-sulfate	$C_6H_8O_9S$	0.951	254.98219	$[M-H]^-$	0.54	1.94	0.0051	0.1173	Down	II
S57	α-Ketoglutaric acid	$C_5H_6O_5$	0.950	145.01465	$[M-H]^-$	0.52	1.97	0.0039	0.1030	Down	I
S58	Ketoleucine	$C_6H_{10}O_3$	3.506	129.05594	$[M-H]^-$	0.44	1.99	0.0028	0.0876	Down	I

S59	Creatine	$C_4H_9N_3O_2$	0.644	132.07773	$[M-H]^+$	0.72	2.01	0.0076	0.1886	Down	I
S60	L-Arginine	$C_6H_{14}N_4O_2$	0.604	175.12033	$[M-H]^+$	0.66	2.05	0.0052	0.1610	Down	I
S61	Valerylcarnitine	$C_{12}H_{23}NO_4$	3.285	246.17201	$[M-H]^+$	0.50	2.10	0.0015	0.0994	Down	I
S62	1-Hydroxypyrene	$C_{16}H_{10}O$	0.600	219.08466	$[M-H]^+$	0.67	2.10	0.0016	0.0995	Down	II
S63	Aconitic acid	$C_6H_6O_6$	1.120	173.00963	$[M-H]^-$	0.35	2.11	0.0014	0.0675	Down	I
S64	Pyridoxamine	$C_8H_{12}N_2O_2$	0.555	169.09598	$[M-H]^+$	0.47	2.13	0.0017	0.1039	Down	I
S65	Guanidinoethyl Sulfonate	$C_3H_9N_3O_3S$	0.634	166.21784	$[M-H]^-$	0.61	2.13	0.0011	0.0646	Down	II
S66	L-Palmitoylcarnitine	$C_{23}H_{45}NO_4$	5.207	400.34491	$[M+H]^+$	0.62	2.14	0.0009	0.0897	Down	I
S67	L-Tryptophan	$C_{11}H_{12}N_2O_2$	3.044	203.08296	$[M-H]^-$	0.66	2.15	0.0010	0.0635	Down	I
S68	N-Acetylvaline	$C_7H_{13}NO_3$	3.267	158.08257	$[M-H]^-$	0.23	2.16	0.0003	0.0437	Down	I
S69	p-coumaric acid	$C_9H_8O_3$	0.986	165.05583	$[M-H]^+$	0.56	2.16	0.0008	0.0818	Down	I
S70	Niacinamide	$C_6H_6N_2O$	0.829	123.05627	$[M-H]^+$	0.57	2.23	0.0006	0.0732	Down	I
S71	Cytosine	$C_4H_5N_3O$	0.703	112.05136	$[M-H]^+$	0.72	2.25	0.0003	0.0612	Down	I

S72	Taurine	$C_2H_7NO_3S$	0.637	126.02293	$[M-H]^+$	0.62	2.25	0.0011	0.0911	Down	I
S73	21-Deoxycortisol	$C_{21}H_{30}O_4$	5.152	347.22427	$[M-H]^+$	0.25	2.26	0.0004	0.0698	Down	I
S74	3-Formylindole	C_9H_7NO	3.044	146.06111	$[M-H]^+$	0.66	2.39	0.0001	0.0450	Down	I
S75	L-Saccharopine	$C_{11}H_{20}N_2O_6$	4.748	277.14569	$[M+H]^+$	0.27	2.41	0.0003	0.0616	Down	II
S76	D17 Prostaglandin E_1	$C_{20}H_{32}O_5$	4.578	351.21826	$[M-H]^-$	0.48	1.74	0.0127	0.1825	Down	I

Note: Measured precursor m/z values can correspond to quasi-molecular ions, adduct ions, or in-source fragment ions acquired in DDA mode；Candidate differential metabolites were selected based on VIP, FC, and nominal $p < 0.05$. Metabolites with FDR-adjusted $p < 0.05$ were considered statistically significant after multiple-testing correction, whereas those with FDR-adjusted $p \geq 0.05$ were treated as exploratory candidates.

Table S7. The candidate differential metabolites in the serum of rats (Model *vs.* H-WJD).

NO.	Name	Formula	t (min)	Measured ion (m/z)	Ion mode	FC	VIP	Nominal. *p*	FDR-adjusted *p*	Regulation	Level
S1	4-Vinylphenol sulfate	$C_8H_8O_4S$	4.319	199.00754	$[M-H]^-$	0.48	2.63	0.0000	0.0075	Down	II
S2	Dimethylurea	$C_3H_8N_2O$	0.976	89.07157	$[M-H]^+$	0.66	2.27	0.0033	0.3602	Down	I

S3	2-Hydroxy-3-methylbutyric acid	$C_5H_{10}O_3$	1.780	117.05611	$[M-H]^-$	0.70	2.10	0.0046	0.2389	Down	I
S4	L-Glutamine	$C_5H_{10}N_2O_3$	0.787	147.07750	$[M-H]^+$	0.73	2.20	0.0079	0.4607	Down	I
S5	Kynurenic acid	$C_{10}H_7NO_3$	3.290	188.03584	$[M+H]^+$	0.58	2.00	0.0094	0.3235	Down	I
S6	γ-Glutamylleucine	$C_{11}H_{20}N_2O_5$	3.140	261.14664	$[M-H]^+$	0.63	2.21	0.0107	0.5009	Down	II
S7	3-Hydroxy-2-methylbutanoic acid	$C_5H_{10}O_3$	2.425	117.05608	$[M-H]^-$	0.79	1.97	0.0114	0.3385	Down	I
S8	Proline-Hydroxyproline	$C_{10}H_{16}N_2O_4$	0.673	229.12022	$[M-H]^+$	0.48	2.18	0.0173	0.5474	Down	II
S9	Monooctyl phthalate	$C_{16}H_{22}O_4$	5.616	277.14508	$[M-H]^-$	0.42	1.89	0.0200	0.3945	Down	II
S10	L-Valine	$C_5H_{11}NO_2$	0.717	118.08708	$[M+H]^+$	0.79	2.10	0.0203	0.5610	Down	I
S11	Glutamylphenylalanine	$C_{14}H_{18}N_2O_5$	3.273	293.11499	$[M-H]^-$	0.81	1.76	0.0288	0.4448	Down	II
S12	N-Acetylleucine	$C_8H_{15}NO_3$	3.699	174.11382	$[M-H]^+$	0.63	1.91	0.0298	0.6255	Down	I
S13	D-Fructose	$C_6H_{12}O_6$	1.022	179.05652	$[M-H]^-$	0.72	1.68	0.0427	0.4835	Down	I
S14	Homovanillic acid sulfate	$C_9H_{10}O_7S$	3.507	261.00806	$[M-H]^-$	16.92	2.57	0.0005	0.1062	Up	II

S15	22α-Hydroxyerythrodiol	$C_{30}H_{50}O_3$	5.974	459.38672	$[M-H]^+$	15.87	2.61	0.0007	0.2086	Up	II
S16	4-Hydroxyhippuric acid	$C_9H_9NO_4$	2.802	194.04634	$[M-H]^-$	6.46	2.38	0.0010	0.1466	Up	I
S17	2-Deoxyribose 5-phosphate	$C_5H_{11}O_7P$	4.747	213.02321	$[M-H]^-$	2.36	2.13	0.0032	0.2066	Up	II
S18	Trigonelline	$C_7H_7NO_2$	0.660	138.05611	$[M+H]^+$	1.77	2.30	0.0033	0.3602	Up	I
S19	3-Hydroxysuberic acid	$C_8H_{14}O_5$	3.719	189.07733	$[M-H]^-$	1.93	2.08	0.0054	0.2485	Up	I
S20	Trimethylamine N-oxide	C_3H_9NO	0.632	76.07633	$[M-H]^+$	2.08	2.14	0.0082	0.4695	Up	I
S21	Vanillic acid 4-O-glucuronide	$C_{14}H_{16}O_{10}$	3.103	343.06778	$[M-H]^-$	6.99	2.13	0.0103	0.3270	Up	II
S22	Taurolithocholic acid	$C_{26}H_{45}NO_5S$	4.502	482.29526	$[M-H]^-$	2.54	1.99	0.0191	0.3927	Up	I
S23	1-Hydroxypyrene	$C_{16}H_{10}O$	0.600	219.08466	$[M-H]^+$	1.24	2.03	0.0197	0.5566	Up	II
S24	3, 5-Tetradecadiencarnitine	$C_{21}H_{37}NO_4$	4.654	368.28241	$[M-H]^+$	1.53	1.93	0.0230	0.5838	Up	II
S25	Dihydrocaffeic acid 3-sulfate	$C_9H_{10}O_7S$	3.303	261.00803	$[M-H]^-$	1.52	1.81	0.0239	0.4217	Up	II

S26	5-Nonyltetrahydro-2-oxo-3-furancarboxylic acid	$C_{14}H_{24}O_4$	4.895	255.16078	$[M-H]^-$	1.77	1.74	0.0285	0.4444	Up	II
S27	Indolelactic acid	$C_{11}H_{11}NO_3$	3.904	204.06708	$[M-H]^-$	1.46	1.67	0.0390	0.4807	Up	I
S28	Isopentenyl diphosphate	$C_5H_{12}O_7P_2$	0.594	244.58092	$[M-H]^-$	1.44	1.69	0.0472	0.4910	Up	II
S29	D17 Prostaglandin E_1	$C_{20}H_{32}O_5$	4.578	351.21826	$[M-H]^-$	1.56	1.63	0.0421	0.4835	Up	I

Note: Measured precursor m/z values can correspond to quasi-molecular ions, adduct ions, or in-source fragment ions acquired in DDA mode；Candidate differential metabolites were selected based on VIP, FC, and nominal $p < 0.05$. Metabolites with FDR-adjusted $p < 0.05$ were considered statistically significant after multiple-testing correction, whereas those with FDR-adjusted $p \geq 0.05$ were treated as exploratory candidates.

Table S8. Metabolomic pathway enrichment data.

Pathway Name	Corresponding metabolites	p	-log(p)	Impact
Nitrogen metabolism	L-Glutamine	0.0193	1.7155	0.0000
Valine, leucine and isoleucine biosynthesis	L-Valine	0.0256	1.5916	0.0000
Arginine biosynthesis	L-Glutamine	0.0445	1.3520	0.0000
Starch and sucrose metabolism	D-Fructose	0.0569	1.2451	0.0046
Terpenoid backbone biosynthesis	Isopentenyl diphosphate	0.0569	1.2451	0.1323
Pantothenate and CoA biosynthesis	L-Valine	0.0569	1.2451	0.1857
Fructose and mannose metabolism	D-Fructose	0.0630	1.2004	0.0000
Pentose phosphate pathway	2-Deoxy-D-ribose 5-phosphate	0.0722	1.1414	0.0861
Galactose metabolism	D-Fructose	0.0843	1.0740	0.0000
Alanine, aspartate and glutamate metabolism	L-Glutamine	0.0873	1.0588	0.1138
Glyoxylate and dicarboxylate metabolism	L-Glutamine	0.0993	1.0031	0.0000

Pyrimidine metabolism	L-Glutamine	0.1199	0.9211	0.0000
Valine, leucine and isoleucine degradation	L-Valine	0.1228	0.9107	0.0000
Amino sugar and nucleotide sugar metabolism	D-Fructose	0.1287	0.8906	0.0000
Purine metabolism	L-Glutamine	0.2095	0.6789	0.0000

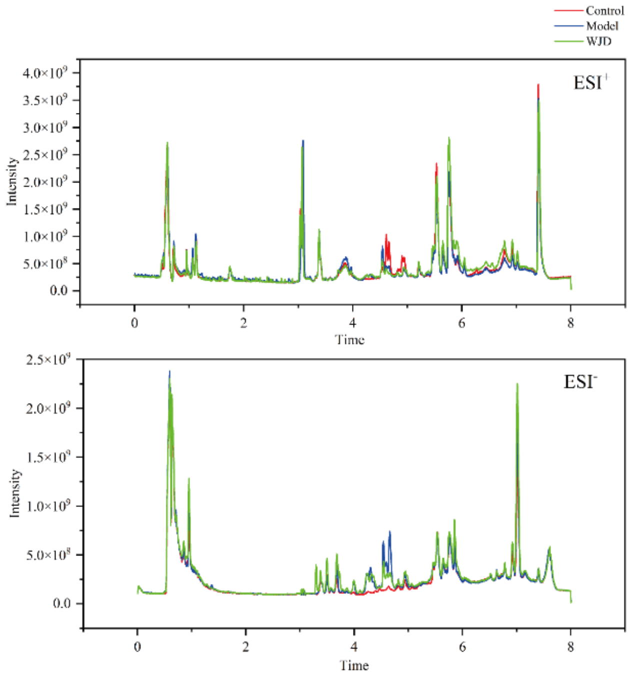

FigureS2. Total ion chromatograms (TIC) of serum metabolites in Control, Model and WJD groups under positive and negative ion modes.

6 Identification of Migratory Compounds

6.1 Comparison procedure of blank serum, WJD extract and drug-containing serum

The stock solution of Wenjing Decoction (WJD) was analyzed to characterize its in vitro chemical components. Blank rat serum was tested to eliminate endogenous matrix interference peaks. The data of drug-containing serum after administration were collected for synchronous comparison. All datasets from the three groups were overlapped and matched. The common peaks detected in both WJD stock solution and drug-containing serum but absent in blank serum were identified as prototype components (P) of the decoction absorbed into blood. The chromatographic peaks exclusively found in drug-containing serum but undetectable in WJD stock solution and blank serum were preliminarily judged as in vivo metabolites (M).

Table S9. Identification of prototype components in WJD rats after oral administration of WJD.

No.	Identification	Formula	Ion mode	t(s)	Measured ion (m/z)	Error(ppm)	Level
P1	Protocatechuic acid	$C_7H_6O_4$	$[M-H]^-$	214.8	153.0192	0.9	I
P2	Syringic acid	$C_9H_{10}O_5$	$[M-H]^-$	219.8	197.0452	1.1	II
P3	Homovanillic acid	$C_9H_{10}O_4$	$[M-H]^-$	226.6	181.0503	1.5	I
P4	Tropone	C_7H_6O	$[M+H]^+$	227.2	107.0493	1.4	I
P5	2,4-Dimethylphenol	$C_8H_{10}O$	$[M-H]^-$	227.7	121.0657	1.5	II
P6	4'-Hydroxyacetophenone	$C_8H_8O_2$	$[M-H]^-$	227.7	135.0449	1.5	I

P7	5-Hydroxy-7-methoxy-6-methylflavone	$C_{17}H_{14}O_4$	$[M-H]^-$	228.0	281.0815	1.5	II
P8	5-Hydroxy-3',4',7-trimethoxyflavone	$C_{18}H_{16}O_6$	$[M-H]^-$	228.5	327.0870	1.5	II
P9	Protocatechualdehyde	$C_7H_6O_3$	$[M-H]^-$	242.0	137.0242	0.1	II
P10	3-Hydroxy-4-methoxyacetophenone	$C_9H_{10}O_3$	$[M+H]^+$	251.8	167.0705	1.4	I
P11	4-Hydroxybenzoic acid	$C_7H_6O_3$	$[M-H]^-$	256.6	137.0242	1.3	I
P12	Albiflorin	$C_{23}H_{28}O_{11}$	$[M+H]^+$	258.9	481.1707	0.6	I
P13	Phthalic acid	$C_8H_6O_4$	$[M-H]^-$	260.6	165.0109	1.7	I
P14	Ethyl gallate	$C_9H_{10}O_5$	$[M-H]^-$	261.7	197.0452	1.6	II
P15	Methyl 3,4-dihydroxybenzoate	$C_8H_8O_4$	$[M-H]^-$	262.3	167.0347	1.9	II
P16	Caffeic acid	$C_9H_8O_4$	$[M-H]^-$	269.7	179.0347	1.3	I
P17	Brevifolincarboxylic acid	$C_{13}H_8O_8$	$[M-H]^-$	272.3	291.0141	2.2	II
P18	4-Hydroxybenzyl alcohol	$C_7H_8O_2$	$[M-H]^-$	273.5	123.0449	1.9	I
P19	Salsolinol	$C_{10}H_{13}NO_2$	$[M+H]^+$	275.6	180.1021	0.9	II

P20	Lonicerin	$C_{27}H_{30}O_{15}$	$[M-H]^-$	279.3	593.1500	1.9	I
P21	3,4-Dihydroxyhydrocinnamic acid	$C_9H_{10}O_4$	$[M-H]^-$	279.3	181.0503	1.7	I
P22	Desaminotyrosine	$C_9H_{10}O_3$	$[M-H]^-$	284.8	165.0554	1.6	I
P23	3',5'-Dimethoxy-4'-hydroxyacetophenone	$C_{10}H_{12}O_4$	$[M-H]^-$	286.3	195.0659	1.8	I
P24	2-Hydroxy-6-methoxybenzoic acid	$C_8H_8O_4$	$[M-H]^-$	298.6	167.0346	2.1	I
P25	Methyl isovanillate	$C_9H_{10}O_4$	$[M-H]^-$	298.7	181.0503	1.7	I
P26	5,7-Dihydroxycoumarin	$C_9H_6O_4$	$[M-H]^-$	299.8	177.0190	1.6	I
P27	3-Hydroxycinnamic acid	$C_9H_8O_3$	$[M-H]^-$	301.5	163.0398	1.4	I
P28	Liquiritin	$C_{21}H_{22}O_9$	$[M-H]^-$	315.2	417.1183	0.6	II
P29	2',4'-Dihydroxyacetophenone	$C_8H_8O_3$	$[M-H]^-$	320.0	151.0398	1.6	I
P30	trans-Ferulic acid	$C_{10}H_{10}O_4$	$[M-H]^-$	320.2	193.0503	1.9	I
P31	Ononin	$C_{22}H_{22}O_9$	$[M+H]^+$	322.6	431.1340	0.7	I
P32	Liquiritigenin	$C_{15}H_{12}O_4$	$[M+H]^+$	342.7	257.0811	1.0	I

P33	Umbelliferone	$C_9H_6O_3$	$[M+H]^+$	344.5	163.0392	1.2	I
P34	4,4'-Di-O-methylellagic acid	$C_{16}H_{10}O_8$	$[M-H]^-$	347.3	329.0299	1.4	II
P35	Benzoylpaeoniflorin	$C_{30}H_{32}O_{12}$	$[M-H]^-$	354.7	583.1811	1.6	I
P36	2-Methoxycinnamic acid	$C_{10}H_{10}O_3$	$[M-H]^-$	358.1	177.0554	1.8	I
P37	Ethyl 4-hydroxybenzoate	$C_9H_{10}O_3$	$[M-H]^-$	369.6	165.0555	1.4	I
P38	Pratol	$C_{16}H_{12}O_4$	$[M+H]^+$	385.5	269.0812	1.3	I
P39	Elemicin	$C_{12}H_{16}O_3$	$[M-H]^-$	386.4	207.1023	1.8	II
P40	5,7-dihydroxy-2-phenylchroman-4-one	$C_{15}H_{12}O_4$	$[M-H]^-$	403.4	255.0659	1.4	I
P41	Gweicurculactone	$C_{15}H_{16}O_2$	$[M+H]^+$	435.4	229.1226	1.4	I
P42	Angeolide	$C_{24}H_{28}O_4$	$[M+H]^+$	444.8	381.2067	1.6	I
P43	Levistilide A	$C_{24}H_{28}O_4$	$[M+H]^+$	470.6	381.2065	1.1	I
P44	Gypsogenin 3-O-β-D-glucuronide	$C_{36}H_{54}O_{10}$	$[M-H]^-$	531.8	645.3632	2.1	II

Table S10. Identification of metabolic components in WJD rats after oral administration of WJD.

No.	Identification	Formula	Ion mode	t(s)	Measured ion (m/z)	Error(ppm)	Level
M1	4-Hydroxyphenylpyruvic acid	$C_9H_8O_4$	$[M-H]^-$	181.1	179.0346	2.0	II
M2	5-Hydroxyisovanillic acid	$C_8H_8O_5$	$[M-H]^-$	236.6	183.0294	2.5	II
M3	Saponarin	$C_{27}H_{30}O_{15}$	$[M-H]^-$	251.8	593.1497	1.8	II
M4	Eriodictyol 7-O-glucuronide	$C_{21}H_{20}O_{12}$	$[M-H]^-$	254.2	463.0871	2.3	II
M5	Curcumenol	$C_{15}H_{22}O_2$	$[M+H]^+$	261.7	235.1696	2.0	II
M6	Veratric acid	$C_9H_{10}O_4$	$[M-H]^-$	299.1	181.0502	2.2	I
M7	Homoorientin	$C_{21}H_{20}O_{11}$	$[M+NH_4]^+$	337.1	466.1351	1.5	II
M8	7,4'-Dihydroxyflavone	$C_{15}H_{10}O_4$	$[M-H]^-$	344.2	253.0502	1.7	I
M9	Ethyl p-coumarate	$C_{11}H_{12}O_3$	$[M-H]^-$	351.4	191.0710	2.0	II
M10	Apigenin 7-O-glucuronide	$C_{21}H_{18}O_{11}$	$[M+H]^+$	356.3	447.0930	0.2	II
M11	1,5-Diferuloylquinic acid	$C_{27}H_{28}O_{12}$	$[M-H]^-$	356.6	543.1496	2.4	II

M12	Vitexin	$C_{21}H_{20}O_{10}$	$[M+H]^+$	359.7	433.1137	0.1	II
M13	Anthragallol	$C_{14}H_8O_5$	$[M-H]^-$	377.8	255.0294	2.3	II
M14	Oroxylin A-7-O-β-D-glucuronide	$C_{22}H_{20}O_{11}$	$[M+H]^+$	381.9	461.1085	0.2	II
M15	Apigenin-7-O-glucoside	$C_{21}H_{20}O_{10}$	$[M-H]^-$	385.9	431.0975	1.9	II
M16	4,5-Di-O-caffeoylquinic acid methyl ester	$C_{26}H_{26}O_{12}$	$[M-H]^-$	394.3	529.1343	1.5	II
M17	Robinetin	$C_{15}H_{10}O_7$	$[M-H]^-$	425.6	301.0380	3.1	II
M18	22α-Hydroxyerythrodiol	$C_{30}H_{50}O_3$	$[M+H]^+$	548.8	459.3837	1.2	II

pharmaceuticals

7 Network pharmacology

Table S11. Network pharmacology targets.

NO.	Target	Degree	Eigenvector
1	AKT1	196	0.1736
2	TNF	192	0.1656
3	MMP9	158	0.1564
4	EGFR	162	0.1562
5	ESR1	160	0.1542
6	MAPK3	150	0.1444

Table S12. Topological analysis parameters.

NO.	Target	Degree	Eigenvector
1	ALB	200	0.1671
2	AKT1	196	0.1736
3	TNF	192	0.1656
4	TP53	166	0.1619
5	BCL2	164	0.1602
6	SRC	162	0.1516
7	CASP3	162	0.1606
8	EGFR	162	0.1562
9	ESR1	160	0.1542

10	MMP9	158	0.1564
11	PTGS2	158	0.1469
12	STAT3	158	0.1584
13	NFKB1	154	0.1547
14	HSP90AA1	152	0.1487
15	MAPK3	150	0.1444
16	CCND1	142	0.1479
17	PPARG	140	0.1418
18	ERBB2	134	0.1379
19	CXCL8	124	0.1245
20	MMP2	124	0.1324
21	FGF2	120	0.1283
22	ICAM1	118	0.1210
23	MTOR	116	0.1309
24	KDR	114	0.1254
25	BCL2L1	110	0.1256
26	MDM2	110	0.1219
27	PARP1	106	0.1199
28	ACE	102	0.0939
29	AR	100	0.1104

30	IL2	98	0.1124
31	BRAF	98	0.1150
32	PIK3CA	96	0.1042
33	KIT	96	0.1115
34	NOS3	94	0.0936
35	PGR	94	0.1055
36	MAP2K1	92	0.1102
37	SERPINE1	92	0.0955
38	IGF1R	90	0.1063
39	NR3C1	90	0.0948
40	NRAS	88	0.0973
41	CASP8	88	0.1062
42	HMOX1	88	0.0997
43	NFE2L2	86	0.0989
44	MET	86	0.1030
45	TERT	84	0.1008
46	PPARA	84	0.0897
47	ABCB1	84	0.0891
48	PLAU	82	0.0926
49	CDK4	82	0.0973
50	MPO	82	0.0827

51	CDK2	80	0.0921
52	LGALS3	80	0.0938
53	FLT1	78	0.0870
54	HNF4A	78	0.0843
55	CYP3A4	76	0.0587
56	ABCG2	76	0.0801
57	ITGB3	70	0.0702
58	FGFR1	70	0.0814
59	DNMT1	70	0.0845
60	ESR2	70	0.0812
61	CDK1	68	0.0801
62	CYP1A1	68	0.0619
63	CDK6	66	0.0778
64	MMP1	66	0.0815
65	RAF1	64	0.0767
66	MMP7	62	0.0757
67	SELP	62	0.0610
68	PTGS1	60	0.0573
69	NOS2	60	0.0671
70	AURKA	60	0.0683

71	XDH	56	0.0608
72	BRD4	54	0.0646
73	ADRB2	54	0.0508
74	RXRA	50	0.0470
75	MMP13	50	0.0623
76	F3	50	0.0505
77	ADAM17	48	0.0571
78	PGF	46	0.0503
79	PIK3CG	46	0.0599
80	ITGB2	46	0.0502
81	TEK	46	0.0540
82	PPARD	46	0.0567
83	NOS1	46	0.0480
84	TYMS	46	0.0464
85	PLK1	44	0.0489
86	NTRK2	44	0.0485
87	HDAC3	42	0.0479
88	FLT4	40	0.0478
89	TRPV1	40	0.0408
90	COL18A1	40	0.0401
91	CDC25C	40	0.0463

92	MME	40	0.0419
93	F2	40	0.0293
94	CYP2C19	38	0.0201
95	PLA2G4A	36	0.0348
96	NR3C2	36	0.0322
97	CYP1B1	36	0.0255
98	ALOX5	34	0.0336
99	CYP17A1	32	0.0196
100	PSEN1	30	0.0295
101	CYP2D6	30	0.0176
102	NR1I3	30	0.0211
103	CYP2A6	30	0.0160
104	TTR	28	0.0261
105	KCNH2	28	0.0187
106	AKR1B1	26	0.0313
107	CFTR	26	0.0273
108	SHBG	22	0.0222
109	CNR1	22	0.0260
110	EDNRA	22	0.0217
111	EPHX1	22	0.0118

112	ITGAL	20	0.0213
113	TBXAS1	20	0.0086
114	F10	20	0.0122
115	AVPR2	20	0.0162
116	EPHB4	18	0.0170
117	PDE5A	18	0.0169
118	F9	16	0.0097
119	TACR1	16	0.0109
120	ODC1	14	0.0177
121	AHR	14	0.0119
122	KCNJ5	14	0.0027
123	CACNA1C	14	0.0065
124	CYP11B2	14	0.0061
125	LGALS1	12	0.0169
126	SLC6A2	12	0.0068
127	PSEN2	12	0.0092
128	CETP	12	0.0112
129	HTR3A	10	0.0046
130	ALOX12	10	0.0058
131	HSD11B2	10	0.0049
132	SCN5A	8	0.0036

133	DBH	6	0.0012
134	HTR4	4	0.0034
135	DHCR7	4	0.0015
136	MYLK	4	0.0037

Table S13. Degree values of core compounds.

NO.	Compend name	Degree
1	7,4'-Dihydroxyflavone	36
2	Pratol	33
3	Robinetin	31
4	Liquiritigenin	28
5	Ethyl p-coumarate	26
6	Albiflorin	21
7	Ferulic acid	20
8	Caffeic acid	18
9	Levistolide A	18
10	Umbelliferone	17
